# Inhibition of Src signaling induces autophagic killing of *Toxoplasma gondii* via PTEN-mediated deactivation of Akt

**DOI:** 10.1371/journal.ppat.1012907

**Published:** 2025-01-27

**Authors:** Alyssa Hubal, Anusha Vendhoti, Charles N. Shaffer, Sarah Vos, Yalitza Lopez Corcino, Carlos S. Subauste

**Affiliations:** 1 Department of Pathology, Case Western Reserve University, Cleveland, Ohio, United States of America; 2 Division of Infectious Diseases and HIV Medicine, Department of Medicine, Case Western Reserve University, Cleveland, Ohio, United States of America; UTSW: The University of Texas Southwestern Medical Center, UNITED STATES OF AMERICA

## Abstract

The intracellular protozoan *Toxoplasma gondii* manipulates host cell signaling to avoid targeting by autophagosomes and lysosomal degradation. Epidermal Growth Factor Receptor (EGFR) is a mediator of this survival strategy. However, EGFR expression is limited in the brain and retina, organs affected in toxoplasmosis. This raises the possibility that *T*. *gondii* activates a signaling mechanism independently of EGFR to avoid autophagic targeting. We report *T*. *gondii* activates Src to promote parasite survival even in cells that lack EGFR. Blockade of Src triggered LC3 and LAMP-1 recruitment around the parasitophorous vacuole (PV) and parasite killing dependent on the autophagy protein, ULK1, and lysosomal enzymes. Src promoted PI3K activation and recruitment of activated Akt to the PV membrane. *T*. *gondii* promoted Src association with PTEN, and PTEN phosphorylation at Y240, S380, T382, and T383, hallmarks of an inactive PTEN conformation known to maintain Akt activation. Blockade of parasite killing was dependent of activated Akt. Src knockdown or treatment with the Src family kinase inhibitor, Saracatinib, impaired these events, leading to PTEN accumulation around the PV and a reduction in activated Akt recruitment at this site. Saracatinib treatment in mice with pre-established cerebral and ocular toxoplasmosis promoted PTEN recruitment around tachyzoites in neural tissue impairing recruitment of activated Akt, profoundly reducing parasite load and neural histopathology that were dependent of the autophagy protein, Beclin 1. Our studies uncovered an EGFR-independent pathway activated by *T*. *gondii* that enables its survival and is central to the development of neural toxoplasmosis.

## Author summary

*Toxoplasma gondii*, a causative agent of retinitis and encephalitis, survives within host cells by avoiding autophagy-dependent degradation by the lysosome. While *T*. *gondii* activates EGFR to avoid autophagic killing, EGFR expression is limited in neural tissue. We uncovered that, independently of EGFR, *T*. *gondii* activates the ubiquitous molecule, Src, resulting in PTEN inhibition and decoration of the parasite-containing vacuole with activated Akt (negative regulator of autophagy). Inhibition of Src impaired this cascade, leading to autophagic killing of *T*. *gondii* demonstrated by autophagosomal and lysosomal marker recruitment and parasite killing dependent on ULK1 and lysosomal enzymes. The Src family kinase inhibitor, Saracatinib, induced PTEN recruitment around parasites in neural tissue and impaired recruitment of activated Akt, causing a striking reduction in parasite load and histopathology in mice with ocular and cerebral toxoplasmosis. Autophagy-deficient mice treated with Saracatinib did not have improved parasite load or histopathology supporting the pivotal role of this pathway for parasite survival and development of toxoplasmosis.

## Introduction

Intracellular pathogens utilize a variety of strategies to manipulate host cells to establish a niche that ensures their survival and proliferation [1–4]. The study of these strategies not only addresses a fundamental aspect of host-pathogen interactions but may also translate into identification of new approaches to treat these infections. The obligate intracellular protozoan, *Toxoplasma gondii*, is an excellent example of a pathogen that survives within the host by deploying strategies that include avoidance of host cell-autonomous mechanisms of defense, manipulation of signal transduction in the host to affect the immune response, and blocking apoptosis of infected cells [5–7]. Indeed, this highly successful pathogen causes chronic infection in approximately 30% of the world population [8]. *T*. *gondii* is important clinically because it is a major cause of infectious retinitis worldwide, can cause encephalitis in immunosuppressed individuals and lead to congenital toxoplasmosis. While antibiotics are available against toxoplasmosis, there is no evidence that they positively affect visual outcome or disease recurrence in the case of ocular toxoplasmosis [9,10]. In addition, antimicrobial agents used for the treatment of cerebral or ocular toxoplasmosis can result in significant side-effects [11].

*T*. *gondii* resides within host cells in a parasitophorous vacuole (PV) that must avoid lysosomal degradation mediated by macroautophagy [5,7]. Macroautophagy, herein referred to as autophagy**,** is a process in which cellular cargo is directed to the lysosomal compartment for degradation [12,13]. This process initiates with the formation of the phagophore, a precursor of the double-membrane autophagosome, which is necessary for the sequestration of cargo, such as intracellular pathogens [14,15]. The phagophore elongates around the sequestered cargo as a result of the assembly of core autophagy machinery proteins that function as a ubiquitin-like conjugation system [15,16]. This results in the formation of a double membrane autophagosome that encircles the cargo which is followed by fusion with lysosomes and cargo degradation [17].

Autophagy is a constitutive process indicating that *T*. *gondii* must employ strategies to avoid targeting by autophagosomes. Indeed, the parasite activates EGFR within the infected host cell to avoid targeting by autophagy [7,18–20]. Inhibition of EGFR signaling in EGFR+ cells either by expression of dominant negative (DN) EGFR or by treatment with EGFR tyrosine kinase inhibitors (TKI) induces autophagic killing of *T*. *gondii* [18,20,21]. Moreover, treatment of mice with ocular and cerebral toxoplasmosis with Gefitinib, an EGFR TKI, results in partial reduction in parasite load and histopathology in the eye and brain that are dependent on Beclin 1 [20]. Despite *in vitro* and *in vivo* studies supporting the relevance of EGFR activation as a strategy for *T*. *gondii* survival, it is important to emphasize that the expression of EGFR is not widespread. In this regard, expression of EGFR in normal adult neural tissue (the main site affected in toxoplasmosis) is moderate and restricted to areas such as the EGF subventricular zone [22]. This likely explains why EGFR inhibition only causes a moderate reduction in parasite load and histopathology in the eye and brain and would point towards the existence of a mechanism to avoid autophagic targeting that allows parasite survival in cells that lack EGFR.

*T*. *gondii* causes PKCα/β-dependent sustained activation of the ubiquitous signaling molecule, Src [20]. We report that Src plays a pivotal role in protecting *T*. *gondii* from autophagic killing in a manner that is independent of the presence of EGFR in host cells. *T*. *gondii* activated Src even in mammalian cells that lack EGFR or express DN EGFR mutant known to ablate EGFR signaling. In *T*. *gondii*-infected cells, Src associated with Phosphatase and Tensin Homolog (PTEN) which was maintained in an inactive state. This enabled sustained activation of Akt at the level of the PV membrane (PVM) and blockade of autophagic targeting. Src knockdown or treatment with Saracatinib, a potent and orally available Src kinase inhibitor, impaired Src-PTEN association, causing PTEN recruitment around the PV and impairing Akt activation at the level of the PVM which resulted in killing of *T*. *gondii*. Treatment with Saracatinib in mice with pre-established ocular and cerebral toxoplasmosis revealed that the Src-PTEN-Akt cascade is of critical importance *in vivo*. Saracatinib caused a profound decrease in parasite load in the eye and brain as well as marked reduction in histopathology that were dependent on the autophagy protein, Beclin 1. These studies identified a signaling mechanism utilized by *T*. *gondii* to ensure its survival that is not restricted to the presence of EGFR and indicate that targeting this pathway leads to a striking control of toxoplasmosis.

## Results

### *T*. *gondii* activates Src in host cells that lack functional EGFR signaling and utilizes Src to promote parasite survival

**Fig 1:**
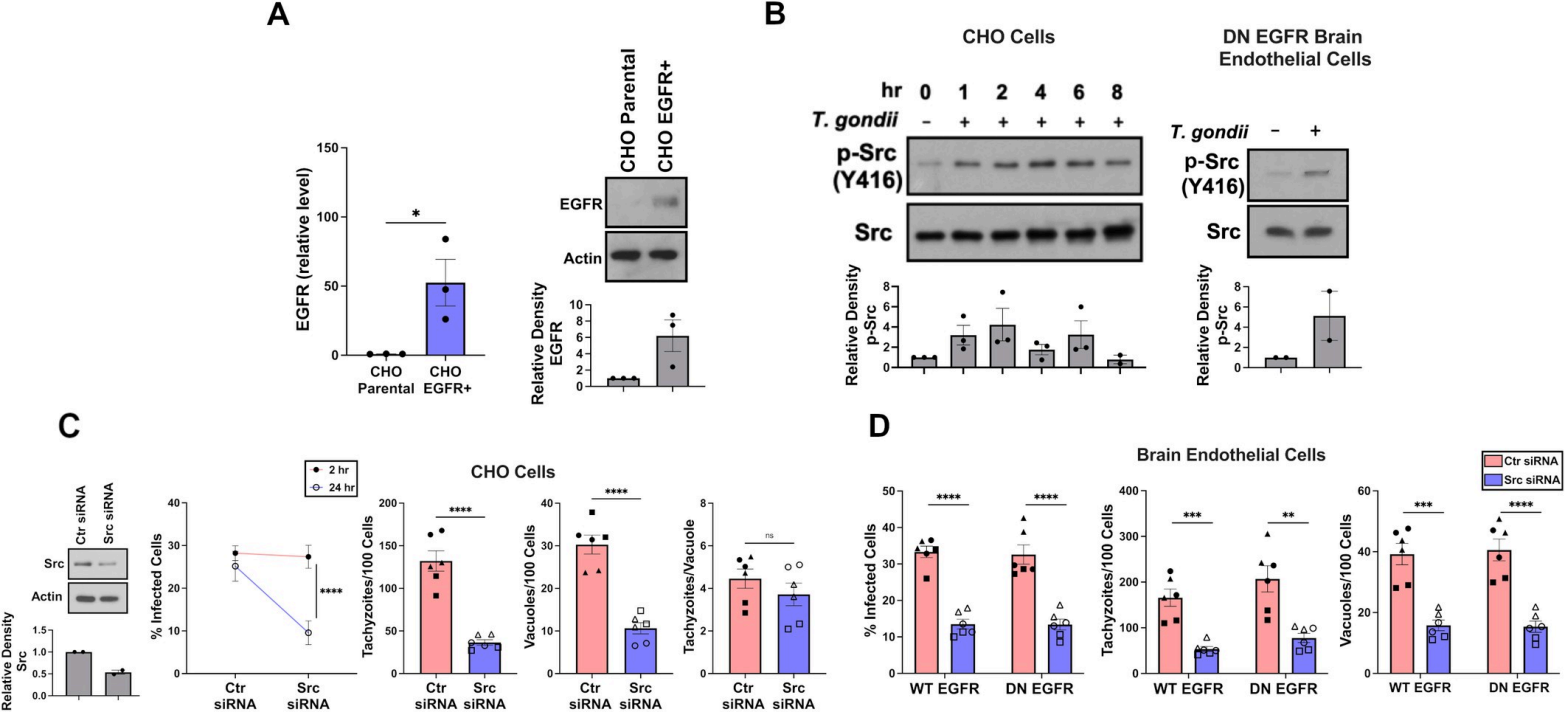
Inhibition of Src triggers killing of *T*. *gondii* in cells that lack functional EGFR. (*A*) EGFR mRNA levels in parental CHO cells and CHO cells stably transfected to express EGFR were measured by RT-qPCR using 18S rRNA as internal control. One sample from parental cells was given an arbitrary value of 1 and data are expressed as fold-increase compared to this sample. Graph represents mean + SEM from 3 independent samples per cell type. Cell lysates were probed for EGFR and actin. Relative density of EGFR was obtained by normalization relative to the parental CHO sample. Densitometry graph represents mean + SEM from 3 independent experiments. (*B*) CHO cells and primary mouse brain endothelial cells that express dominant negative (DN) EGFR were challenged with RH *T*. *gondii*. CHO cells were lysed at the indicated times whereas brain endothelial cells were lysed after 2 h. Lysates were probed for Src and phospho-Y416 Src. Relative density of phospho-Src was obtained by normalization to total Src followed by normalization relative to the uninfected sample. Relative density of phospho-Src for uninfected sample was given a value of 1. Data are shown as mean + SEM from 2 or 3 independent experiments. (*C*, *D*) CHO cells (*C*), and primary mouse brain endothelial cells expressing WT EGFR or DN EGFR (*D*) transfected with Ctr or Src siRNA were challenged with RH *T*. *gondii* and examined at 2 and 24 h. Results are shown as mean ± SEM. The graphs show data from 6 independent monolayers pooled from 3 different experiments. Data points are shown as circles, triangles, or squares corresponding to individual monolayers within each experiment. All significance was determined using two-tailed, unpaired Student’s *t* test comparing six independent monolayers per group (**p<0.01,***p<0.001, ****p<0.0001).

In contrast to EGFR, Src is a ubiquitously expressed signaling molecule [23]. We sought to examine whether *T*. *gondii* activates Src in cells that lack EGFR. Parental CHO cells, confirmed to be devoid of EGFR as assessed by real-time quantitative PCR and immunoblot (Fig 1A), exhibited Src phosphorylation at Tyr416 (marker of Src activation) after infection with *T*. *gondii* (Fig 1B). Similarly, the parasite enhanced Src Tyr416 phosphorylation in brain endothelial cells that express DN EGFR, a mutant previously proven to ablate EGFR signaling [21] (Fig 1B). Next, we examined whether Src promotes survival of *T*. *gondii* in cells that lack EGFR signaling. Knockdown of Src in CHO cells resulted in a decrease in the percentage of infected cells at 24 h vs 2 hr, reduction of the number of vacuoles per 100 cells (both indicative of toxoplasmacidal activity) and a decrease in the number of tachyzoites per 100 cells at 24 hours post-infection (Fig 1C). Knockdown of Src did not affect the number of tachyzoites per vacuole in the cells that remained infected (Fig 1C) indicating that deficiency of Src induced parasite killing rather than reduce intracellular replication. Knockdown of Src in primary brain endothelial cells that express DN EGFR or WT EGFR resulted in a reduction in percentage of infected cells, vacuoles per 100 cells and number of tachyzoites per 100 cells (Fig 1D).

**Fig 2:**
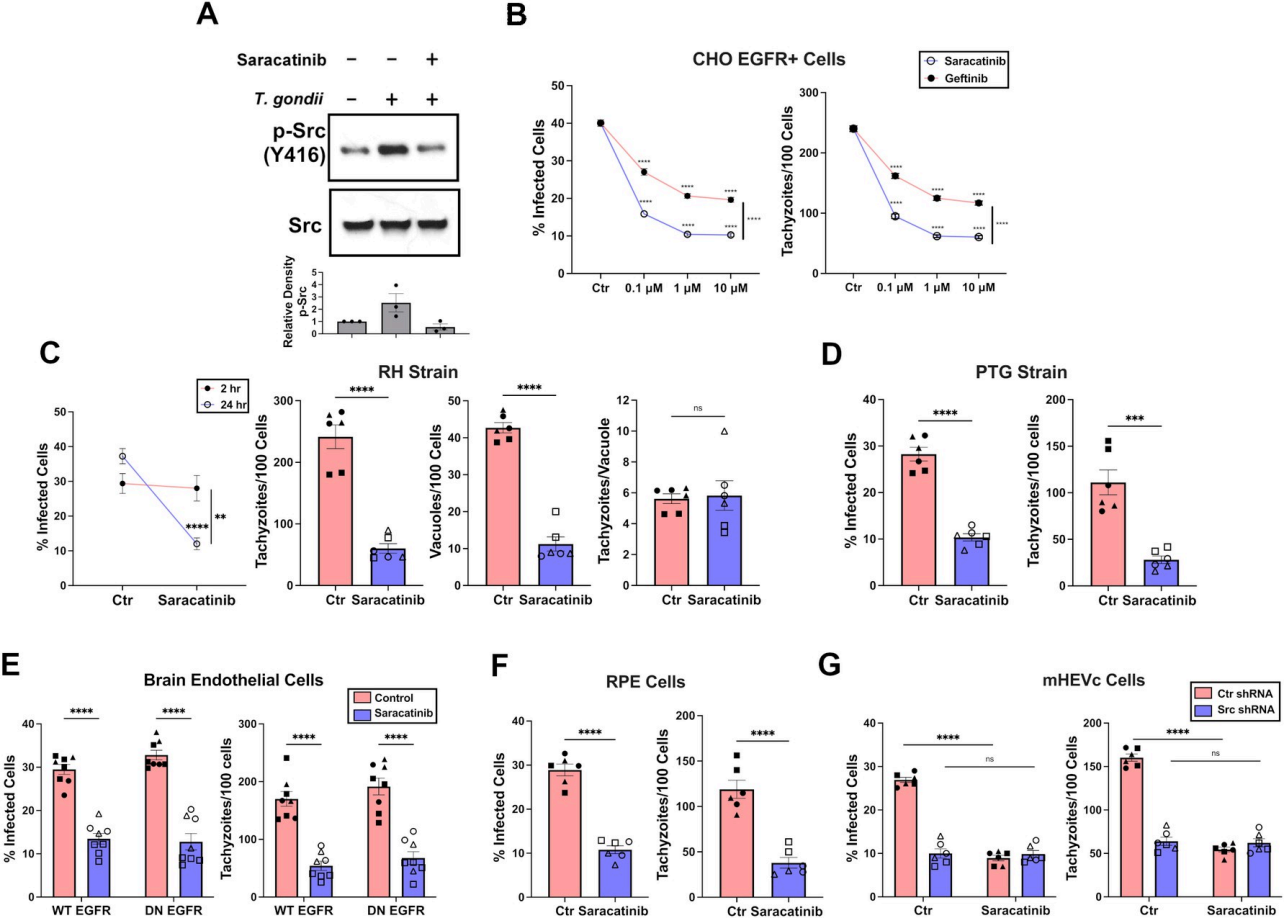
Inhibition of *T*. *gondii*-induced Src signaling triggers parasite killing even in cells that lack functional EGFR. (*A*) CHO cells were challenged with RH *T*. *gondii* and treated with or without Saracatinib (1 μM). Lysates collected at 2 h post-infection were probed for Src and phospho-Y416 Src. Relative density of phospho-Src was normalized to total Src followed by normalization relative to the uninfected sample. Densitometry graph shows mean + SEM from 3 independent experiments. (*B*) EGFR^+^ CHO cells infected with RH *T*. *gondii* were treated with the indicated concentrations of Saracatinib or Gefitinib. Percentages of infection and tachyzoites/100 cells were assessed after 24 h. (*C*, *D*) CHO cells were infected with RH (Type 1) or PTG (Type 2) strains of *T*. *gondii*. Cells were assessed after 2 h and 24 h. (*E*) Primary mouse brain endothelial cells expressing WT or DN EGFR were infected with RH *T*. *gondii* and treated with or without Saracatinib (1 μM). Cells were evaluated at 24 h. (*F*) RPE cells were challenged with RH *T*. *gondii* and treated with or without Saracatinib (1 μM). Percentage of infected cells and number of tachyzoites per 100 cells were determined by light microscopy 24-hr post-infection. (*G*) Mouse endothelial cells (mHEVc) transduced with control shRNA or Src shRNA were incubated with or without Saracatinib (1 μM). Data are shown as mean ± SEM. The graphs show data from 6 independent monolayers pooled from 3 different experiments. Data points are shown as circles, triangles, or squares corresponding to individual monolayers within each experiment. All significance was determined using two-tailed, unpaired Student’s *t* test comparing six independent monolayers per group (***p<0.001, ****p<0.0001).

We further examined the effects of inhibition of Src signaling using Saracatinib, a potent and selective Src family kinase inhibitor [24,25]. Saracatinib markedly impaired *T*. *gondii*-induced Tyr416 phosphorylation of Src in CHO cells (Fig 2A). Moreover, Saracatinib caused a dose-dependent a significant decrease in the percentage of infected cells and tachyzoites per 100 cells (Fig 2B). Those effects were more striking than the effects of Gefitinib, an EGFR family kinase inhibitor, even in CHO cells stably transfected to express EGFR (Fig 2B). Subsequent studies were performed using Saracatinib at 1 μM, a concentration that was previously reported to be non-toxic in various cell lines [25, 26], results that were confirmed in CHO and retinal pigment epithelial (RPE) cells (S1 Fig). Saracatinib-induced toxoplasmacidal activity in CHO cells infected with type I or Type II strains of *T*. *gondii* (Fig 2C and 2D). Saracatinib induced similar effects in brain endothelial cells that expressed WT or non-functional DN EGFR (Fig 2E), and RPE (cells that express native EGFR; Fig 2F). Saracatinib did not affect parasite invasion of host cells (S2A Fig) or parasite replication (S2B Fig). Importantly, Saracatinib acted on target since no toxoplasmacidal activity was detected if cells were made deficient in Src (Fig 2G). Moreover, while Saracatinib can also inhibit Fyn, Yes, and c-Abl [27,28], studies in cells deficient in Src, Yes and Fyn (SYF) and in cells deficient in only Yes and Fyn (SYF + Src) indicate that deficiency in Src, but not deficiency of Yes plus Fyn, affects survival of *T*. *gondii* (S3A Fig). Similarly, inhibition of c-Abl did not affect parasite survival (S3B Fig). Taken together, these findings indicate that Src plays a critical role in promoting survival of *T*. *gondii* independent of the presence of functional EGFR.

### Inhibition of Src in cells deficient in EGFR signaling induces autophagy-mediated killing of *T*. *gondii*

**Fig 3:**
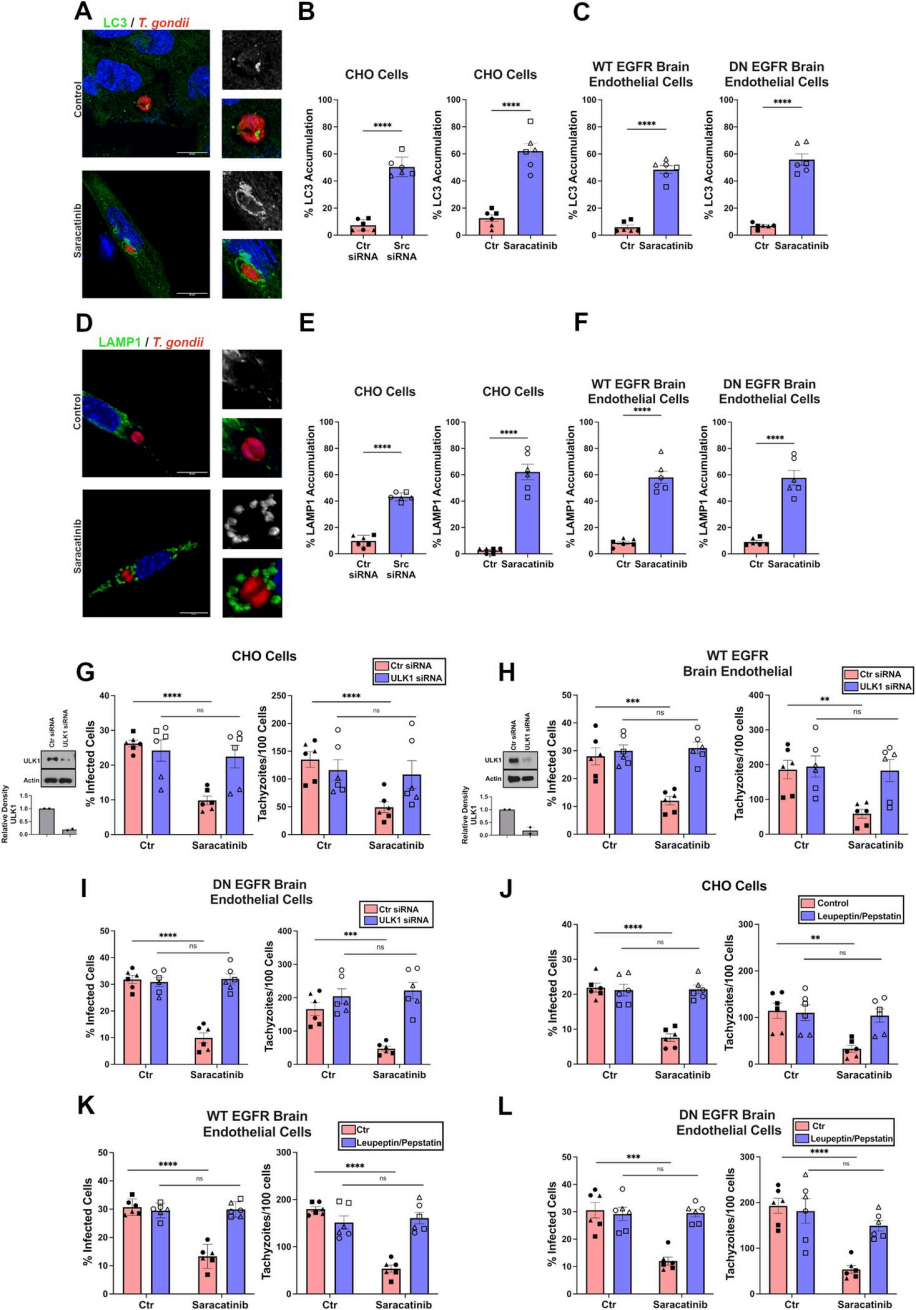
*T*. *gondii* killing induced by Src inhibition is mediated by autophagy. (*A-C*) CHO cells and primary brain endothelial cells expressing WT or DN EGFR were infected with RFP-*T*. *gondii* and treated with Saracatinib 1 μM for 5 hours before staining with an anti-LC3 antibody. Accumulation of LC3 around the parasites was assessed by confocal microscopy. Scale bar, 10 μm. (*D-F*) CHO cells and primary brain endothelial cells expressing WT or DN EGFR were infected with RFP-*T*. *gondii* and treated with Saracatinib 1 μM for 7 hours before staining with anti-LAMP1 antibody. Accumulation of LAMP1 around parasites was assessed as above. *G-I*) CHO cells and primary brain endothelial cells expressing WT or DN EGFR were transfected with control siRNA or ULK1 siRNA. Relative density of ULK1 was normalized to total Actin followed by normalization relative to Ctr siRNA. Densitometry graph shows mean + SEM from 2 independent experiments. Cells were treated with or without Saracatinib and infected with RH *T*. *gondii*. Monolayers were examined at 24 h. (*J-L*) CHO cells and primary brain endothelial cells expressing WT or DN EGFR were infected with RH *T*. *gondii* and incubated with or without Saracatinib. Leupeptin plus pepstatin were added post-infection. Data are shown as mean ± SEM. The graphs show data from 6 independent monolayers pooled from 3 different experiments. Data points are shown as circles, triangles, or squares corresponding to individual monolayers within each experiment. All significance was determined using two-way unpaired Student’s *t* test comparing 6 individual monolayers per group (**p<0.01,***p<0.001,****p<0.0001).

We sought to determine whether Src inhibition triggers autophagy-mediated killing of *T*. *gondii* even in cells lacking functional EGFR. Knockdown of Src or treatment with Saracatinib induced accumulation of the autophagosomal marker LC3 around the parasites in CHO cells and brain endothelial cells that express DN EGFR (Fig 3A–3C). Similarly, accumulation of the lysosomal marker, LAMP1, was observed around intracellular parasites in cells deficient in Src or treated with Saracatinib (Fig 3D–3F). To determine whether killing of *T*. *gondii* occurred through autophagy, we examined the effects of knockdown Unc-51-like kinase 1 (ULK1), an upstream inducer of the autophagy cascade required for autophagosome formation [29]. ULK1-deficient cells were unable to exhibit anti-*T*. *gondii* activity despite treatment with Saracatinib (Fig 3G–3I) or Src knockdown (S4A Fig). To determine if *T*. *gondii* killing was a result of lysosomal degradation, we examined the effects of acid protease inhibitors, Leupeptin and Pepstatin A. Incubation with Leupeptin and Pepstatin A ablated the anti-*T*. *gondii* activity induced by Saracatinib (Fig 3J–3L). Taken together, inhibition of Src induced autophagy-mediated killing of *T*. *gondii* even in cells defective in EGFR signaling.

### Src inhibition in cells deficient in EGFR signaling induces killing of *T*. *gondii* by impairing Akt activation

**Fig 4:**
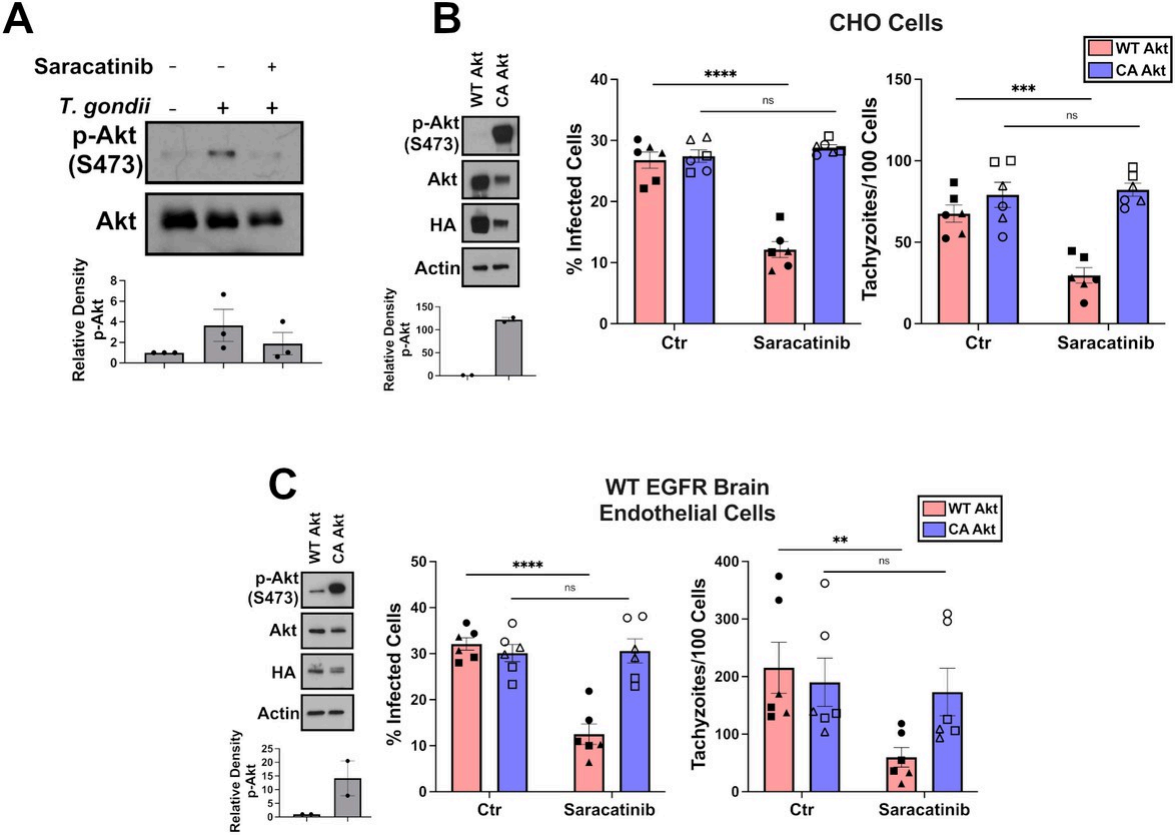
Src inhibition induces *T*. *gondii* killing by impairing Akt activation. (*A*) CHO cells were challenged with RH-*T*. *gondii*. Cell lysates collected at 2 h were probed for Akt and phospho-S473 Akt. Relative density of phospho-Akt was normalized to total Akt and compared to the uninfected sample. Densitometry graph shown as mean + SEM from 3 independent experiments. (*B-C*) CHO cells or primary brain endothelial cells were transfected with plasmids encoding HA-tagged CA- or HA-tagged WT-Akt. Lysates were probed for actin, HA, Akt and phospho-S473 Akt. Cells were challenged with *T*. *gondii* and treated with Saracatinib (1 μM). Cell lysates collected at 2 h were probed for Akt and phospho-S473 Akt. Relative density of phospho-Akt and densitometries were examined as above. Percentages of infected cells and tachyzoites per 100 cells were assessed at 24 h. Data are shown as mean ± SEM. The graphs show data from 6 independent monolayers pooled from 3 different experiments. Data points are shown as circles, triangles, or squares corresponding to individual monolayers within each experiment. Significance was determined using two-way unpaired Student’s *t* test comparing 6 individual monolayers per group (**p<0.01, ***p<0.001, ****p<0.0001).

Akt negatively regulates autophagy [30]. We investigated whether Src acts through Akt to prevent *T*. *gondii* killing in cells deficient in EGFR signaling. *T*. *gondii* infection in CHO cells increased phosphorylation of Akt at the activation site Ser473, an effect that was abrogated by treatment with Saracatinib (Fig 4A). Next, to determine the role of Akt in regulating the killing of *T*. *gondii* induced by Src inhibition, we transfected cells with a plasmid encoding a constitutively active Akt that is directed to membranes by the addition of a Src myristoylation sequence (901 pLNCX myr HA Akt1) or a plasmid encoding a non-myristoylated control as wild-type Akt (903 pLNCX HA Akt1) [31]. Transfection with the myristoylated Akt plasmid (referred to as CA Akt) increased the expression of phospho-S473 Akt in comparison to transfection with non-myristoylated Akt plasmid (referred to as WT Akt) (Fig 4B). Treatment with Saracatinib reduced the percentage of infected cells and the number of tachyzoites per 100 cells in CHO cells transfected with WT Akt (Fig 4B). In contrast, CA Akt transfected CHO cells treated with Saracatinib showed no reduction in percentage of infection or number of tachyzoites per 100 cells in comparison to untreated cells (Fig 4B). These results were confirmed in brain endothelial cells (Fig 4C) as well as in endothelial cells deficient in Src (S4B Fig). Taken together, these results support that inhibition of Src induces parasite killing by impairing Akt activation.

### Inhibition of Src impairs *T*. *gondii*-induced Akt activation in a PTEN-dependent manner

**Fig 5:**
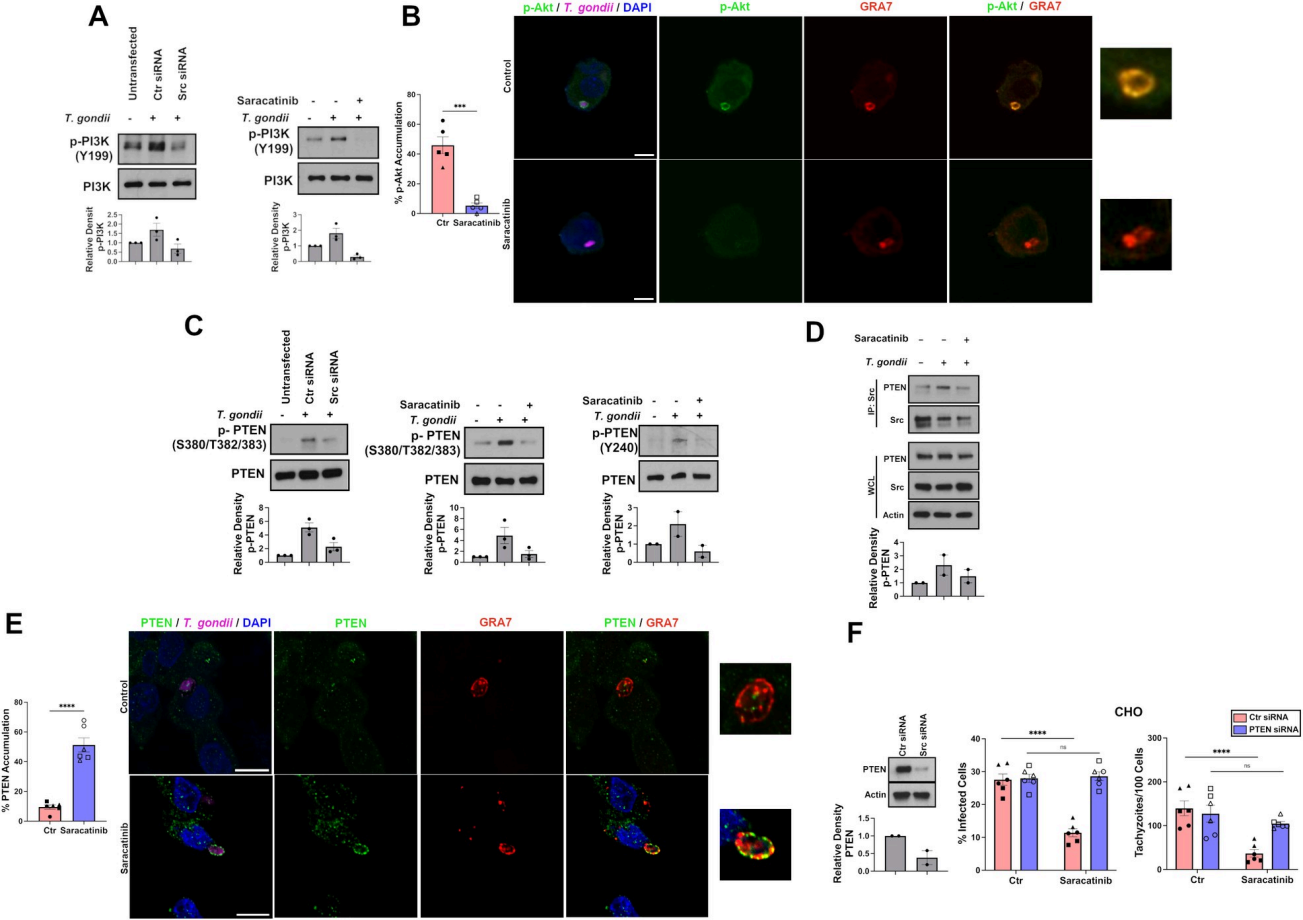
Src impairs the function of PTEN to regulate Akt signaling. (*A*) CHO cells were transfected with control or Src siRNA or treated with or without Saracatinib followed by infection with RH *T*. *gondii*. Cell lysates were obtained at 2 h and were probed for PI3K and phospho-Y199 PI3K. Relative density of phospho-PI3K was normalized to total PI3K and compared to the uninfected sample. Densitometry analysis of blots shown from 3 independent experiments. (*B*) CHO cells infected with RFP-*T*. *gondii* (magenta) and treated with or without Saracatinib. Cells were stained with antibodies against phospho-S73 Akt (green) and GRA7 (red). DAPI (blue) stains the nucleus. Accumulation of phospho-S73 Akt around the parasites was assessed by confocal microscopy. Scale bar, 10 μm. Data are shown as mean ± SEM. The graphs show data from 5 independent monolayers pooled from 3 different experiments. Data points are shown as circles, triangles, or squares corresponding to individual monolayers within each experiment. (*C*) CHO cells were transfected with or without Ctr or Src siRNA or incubated with or without Saracatinib. Cell lysate obtained at 2 h were probed with total PTEN, phospho-S380/T382/383 PTEN or phospho-Y240 PTEN. Densitometry analysis shows means + SEM from 2–3 independent experiments. (*D*) Infected and uninfected CHO cells were subjected to immunoprecipitation using an anti-Src antibody. Whole cell lysates and immunoprecipitated were probed using antibodies against PTEN and Src. Relative density of PTEN in the immunoprecipitate (IP) was obtained by normalization to total Src in the IP followed by normalization relative to the IP from uninfected sample. Relative density of PTEN for uninfected sample was given a value of 1. Densitometry was assessed as above. (*E*) CHO cells infected with RFP-*T*. *gondii* were stained with antibodies against PTEN (green) and GRA7 (red). DAPI (blue) stains the nucleus. Accumulation of PTEN around the parasites was assessed by confocal microscopy. The graphs show data from 6 independent monolayers pooled from 3 different experiments. Data points are shown as circles, triangles, or squares corresponding to individual monolayers within each experiment. (*F*) CHO cells were transfected with control siRNA or PTEN siRNA prior to challenge with RH *T*. *gondii* and incubated with or without Saracatinib. Monolayers were examined at 24 h. Data are shown as mean ± SEM. The graphs show data from 6 independent monolayers pooled from 3 different experiments. Data points are shown as circles, triangles, or squares corresponding to individual monolayers within each experiment. Significance was determined using two-way unpaired Student’s *t* test (***p<0.001, ****p<0.0001).

We conducted studies to identify the mechanism through which inhibition of Src regulates *T*. *gondii*-induced Akt activation. *T*. *gondii* infection in CHO cells increased Y199 phosphorylation of PI3K, a marker of activation of a signaling molecule that increases production of phosphatidylinositols that are key to the activation of Akt (Fig 5A). Y199 phosphorylation of PI3K was inhibited by Src knockdown or treatment with Saracatinib (Fig 5A). Infection with *T*. *gondii* resulted in accumulation of phospho-Ser473 Akt around the parasite (Fig 5B). Expression of activated Akt was associated with the PVM since phospho-Ser473 Akt co-localized with the dense granule protein GRA7 (Fig 5B). Saracatinib inhibited accumulation of phospho-S473 Akt around intracellular parasites (Fig 5B). Thus, *T*. *gondii* promotes Src-dependent expression of activated Akt associated with the PVM.

PTEN acts as a negative regulator of Akt signaling through dephosphorylation of phosphatidylinositols [32]. PTEN binds phospholipids through the C2 domain and functions as a phosphatase [33]. However, phosphorylation at serine and threonine sites (Ser380, Thr382, Thr383) in the regulatory C-terminal tail of PTEN results in interaction between the C-terminal tail and the C2 domain leading to a “closed” inactive conformation [33–35]. In addition, Src phosphorylates Tyr240 in the C2 domain of PTEN limiting the ability of PTEN to dephosphorylate phosphatidylinositols [36,37]. Infection with *T*. *gondii* resulted in increased phosphorylation of PTEN at Tyr240 that was accompanied by an increase in Ser380 and Thr382/383 phosphorylation, indicating a “closed” conformation with impaired phosphatase activity (Fig 5C). Src knockdown or Saracatinib treatment in *T*. *gondii*-infected cells reduced PTEN phosphorylation at Tyr240 as well as at Ser380 and Thr382/383 (Fig 5C). Immunoprecipitation studies indicated an increased association of Src and PTEN in *T*. *gondii*-infected cells, a phenomenon that was impaired by Saracatinib (Fig 5D). Membrane association is a feature of biologically active PTEN [38,39], an event that is impaired by tyrosine phosphorylation [36]. Saracatinib resulted in co-expression of PTEN and GRA7 supporting an association of PTEN to the PVM (Fig 5E). Finally, we examined whether PTEN is required to induce killing of *T*. *gondii* in cells treated with Saracatinib. Cells made deficient in PTEN by transfection with siRNA were no longer able to kill *T*. *gondii* when treated with Saracatinib (Fig 5F). Altogether, *T*. *gondii* induces Src-dependent decoration of PVM with activated Akt, an event that is accompanied by PTEN phosphorylation at amino acid residues indicative of a “closed” inactive conformation of PTEN. In contrast, Src inhibition results in dephosphorylation of these amino acids (markers of PTEN activation), translocation of PTEN around the PV and loss of recruitment of activated Akt to the PVM. PTEN is essential for killing of *T*. *gondii* induced by Src inhibition.

### Saracatinib induces accumulation of PTEN and reduces expression of activated Akt around *T*. *gondii* in neural tissue, as well as confers protection against pre-established ocular and cerebral toxoplasmosis

**Fig 6:**
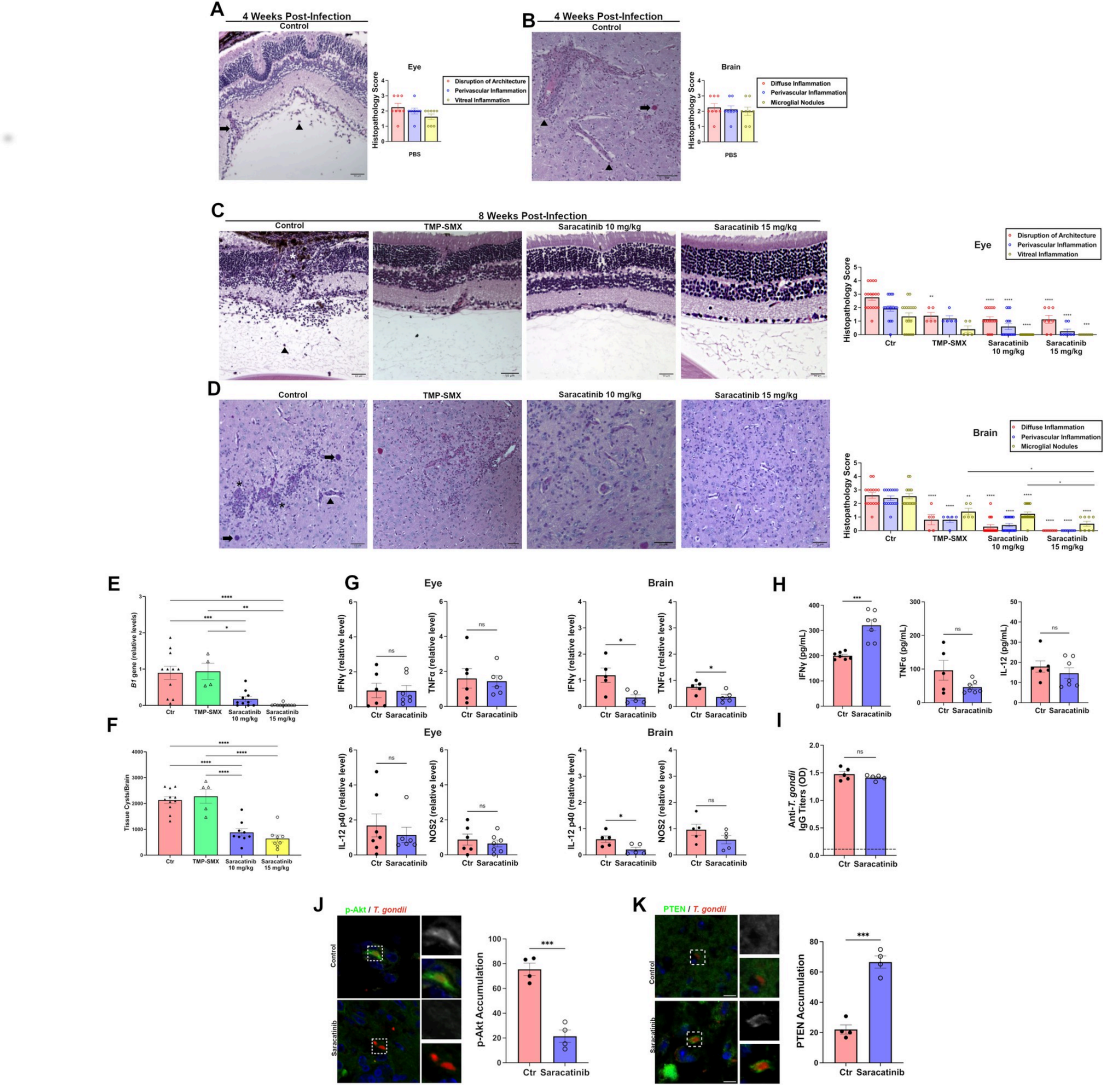
Saracatinib confers protection against ocular and cerebral toxoplasmosis. B6 mice were infected with 10 ME49 *T*. *gondii* tissue cysts for four weeks before treatment with Saracatinib (10 or 15 mg kg^-1^ oral gavage twice per day; 5 days per week), TMP-SMX (120:600 mg kg^-1^ oral gavage once a day; 5 days per week) or vehicle. Mice were euthanized after 4 weeks of treatment (8 weeks total infection). (*A-B*) Representative images of retinas and brains of chronically infected mice after four weeks of infection prior to Saracatinib, TMP-SMX, or vehicle administration. Retina: arrowhead = vitreal inflammation; arrow = perivascular inflammation; asterix = disruption of retinal architecture. Brain: arrowhead = perivascular inflammation; arrow, tissue cyst; asterix; microglial nodule. Scale bar, 50 μm. Data represented as mean ± SEM from 8 mice per group pooled from 2–3 independent experiments. *(C-D)* Representative images of retinas and brains as well as histopathological scoring at 8 weeks post-infection (4 weeks of infection followed by 4 weeks of treatment). Scale bar, 50 μm. Data are shown as mean ± SEM from mice pooled from 2–3 independent experiments: Retina- n = 17 (Ctr), 5 (TMP-SMX), 15 (Saracatinib 10 mg/kg), and 8 (Saracatinib 15 mg/kg). Brain- n = 15 (Ctr), 5 (TMP-SMX), 17 (Saracatinib 10 mg/kg), and 8 (Saracatinib 15 mg/kg). Statistical significance was determined using two-way ANOVA with Tukey multiple comparison test. (*E*) *T*. *gondii B1* gene was examined in the eye qPCR. Levels were compared to those of one control mouse that was given an arbitrary value of 1. Bars represent mean ± SEM pooled from mice pooled from 2–3 independent experiments: n = 11 (Ctr), 4 (TMP-SMX), 10 (Saracatinib 10 mg/kg), and 10 (Saracatinib 15 mg/kg). Statistical significance was determined using one-way ANOVA with Tukey multiple comparison test. (*F*) Number of *T*. *gondii* tissue cysts were counted per brain homogenates. Data are shown as mean ± SEM from mice pooled from 2–3 independent experiments: n = 11 (Ctr), 5 (TMP-SMX), 9 (Saracatinib 10 mg/kg), and 8 (Saracatinib 15 mg/kg). Statistical significance was determined using one-way ANOVA with Tukey multiple comparison test. (*G*). Levels of IL-12 p40, IFN-γ, TNF-α and/or NOS2 mRNA in the eye and brain were measured using RT-qPCR. Data shown as mean ± SEM from mice pooled from 2 independent experiments: Retina- n = 6 Ctr, 7 Saracatinib (IFN-γ), 6 per group (TNF-α), 7 Ctr, 6 Saracatinib (IL-12 p40), and 6 per group (NOS2). Brain- n = 5 per group (IFN-γ, TNF-α, IL-12 p40, NOS2). Statistical significance was determined using two-way unpaired *t* test. *(H)* Serum levels of IFN-γ, TNF-α and IL-12 p40 were measured by ELISA. Data shown as mean ± SEM from mice pooled from 2 independent experiments: n = 7 per group (IFN-γ), 5 Ctr, 7 Saracatinib (TNF-α), and 6 Ctr, 7 Saracatinib (IL-12 p40). Statistical significance was determined using two-way unpaired *t* test. (*I*) Serum titers of anti-*T*. *gondii* IgG were measured by ELISA and are represented as mean ± SEM from 5 mice per group. Results are representative of 2 independent experiments. (*J*, *K*) B6 mice infected with ME49 *T*. *gondii* tissue cysts for 6 weeks were treated with Saracatinib or vehicle for four days. Brain sections were stained with antibodies against *T*. *gondii*, phospho-S473 Akt or PTEN. Scale bar, 10 μm. Data is shown as mean ± SEM from 4 mice. Statistical significance determined using two-way unpaired Student’s *t* Test (*p<0.05, **p<0.01, ***p<0.001, ****p<0.0001).

In contrast to EGFR, Src is highly expressed in the brain and retina [40]. Indeed, immunoblot studies revealed high expression of Src in the brain and retina compared to the spleen, liver and lung of B6 mice (S5 Fig). Saracatinib is a well-tolerated drug that penetrates the blood-brain barrier [27]. Saracatinib is approved for the treatment of idiopathic pulmonary fibrosis [41] and has been used in various clinical trials including in patients with Alzheimer’s and Parkinson’s disease [42,43]. B6 mice infected with tissue cysts of ME49 *T*. *gondii* for four weeks showed histopathology in the eye and brain indicative of ocular and cerebral toxoplasmosis (Fig 6A and 6B). In addition, infected mice had a high tissue cyst burden at this time post-infection (S6 Fig). B6 mice infected for four weeks (with pre-established ocular and cerebral toxoplasmosis) were then treated with Saracatinib for four weeks (10 mg kg^-1^ or 15 mg kg^-1^ twice per day, doses predicted to achieve brain concentrations ~ 0.2 μM) [27], or vehicle. Mice treated with Saracatinib had a profound reduction in parasite load in the eye and brain as well as a marked reduction in histopathology in these organs (Fig 6C–6F). No significant ocular inflammation was detected in 75% of the mice treated with the 15 mg kg^-1^ dose (Fig 6C and 6D). No significant perivascular and diffuse inflammation were detected in the brains of those mice (Fig 6C and 6D). In parallel experiments, mice treated with a high-dose of Trimethoprim-Sulfamethoxazole [44], an antibiotic regimen reported to be equally effective, but better tolerated than Pyrimethamine plus Sulfadiazine in patients with cerebral toxoplasmosis [45,46], exhibited a less pronounced reduction in histopathology in the eye and brain compared to Saracatinib (Fig 6C and 6D). Protection against ocular and cerebral toxoplasmosis in Saracatinib-treated mice was not the result of increased local expression of IFN-γ, TNF-α, IL-12 p40, and NOS2, mediators of resistance against ocular and cerebral toxoplasmosis [47] (Fig 6G). Saracatinib did not affect serum levels of TNF-α and IL-12 p40 and caused a modest increase in serum IFN-γ levels (Fig 6H) consistent with the report that low doses of Saracatinib can enhance IFN-γ production [48]. Anti-*T*. *gondii* IgG levels were unchanged in mice treated with Saracatinib (Fig 6I).

Next, we examined the expression of activated Akt and PTEN *in vivo*. Tachyzoites were present in the brains of mice with cerebral toxoplasmosis and immunofluorescence in brain sections from infected mice that received vehicle demonstrated accumulation of phospho-S473 Akt around tachyzoites (Fig 6J). Saracatinib markedly reduced phospho-S473 Akt accumulation. Moreover, Saracatinib induced recruitment of PTEN around tachyzoites (Fig 6K).

To further explore the effects of Saracatinib, we used chronically infected mice where neural toxoplasmosis had been exacerbated by an immunosuppressive agent. B6 mice infected with ME49 *T*. *gondii* tissue cysts for 3 weeks were given Dexamethasone (0.1 mg kg-1, once per day) for 2 weeks. Examination of brains and eyes revealed that ocular and cerebral toxoplasmosis were exacerbated by Dexamethasone (S7A and S7B Fig). After 2 weeks of Dexamethasone administration, infected mice were treated with either vehicle or Saracatinib (10 mg kg-1 twice per day) for 2 weeks while Dexamethasone administration was continued. Saracatinib significantly reduced histopathology and parasite load in the eye and brain compared to vehicle-treated mice (S7C–S7F Fig). Thus, Saracatinib effectively controlled ocular and cerebral toxoplasmosis in chronically infected mice, even if the animals received an immunosuppressant.

**Fig 7:**
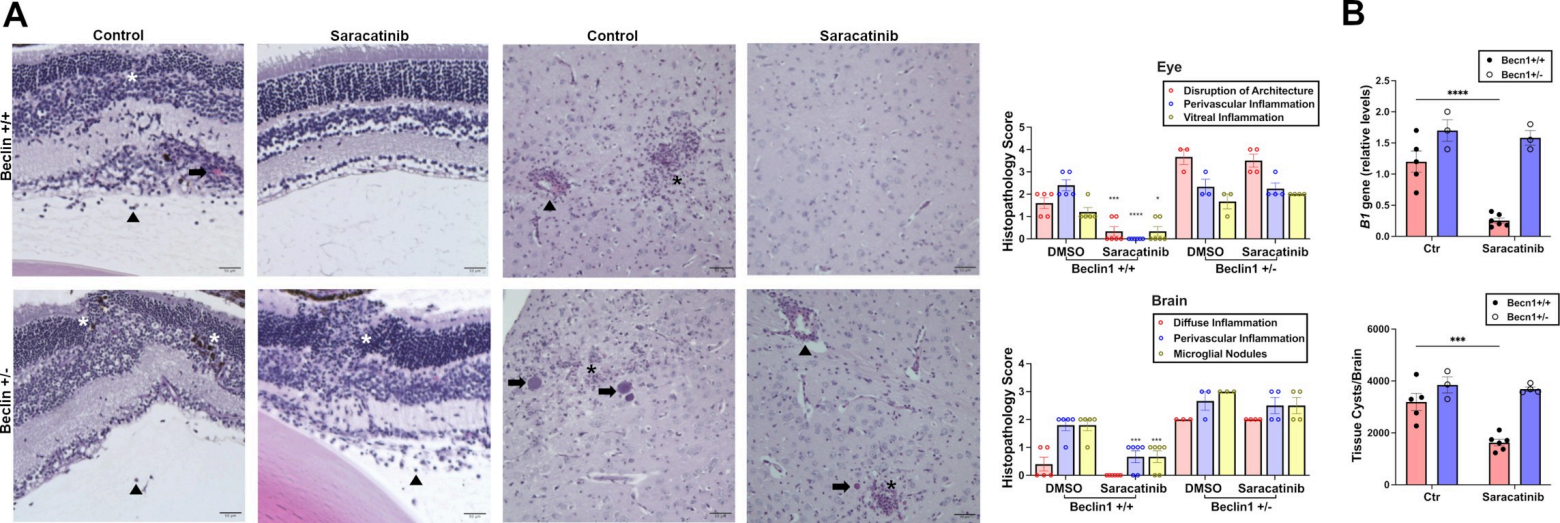
Saracatinib confers protection against ocular and cerebral toxoplasmosis in a Beclin 1-dependent manner. *Becn1*^+/+^ and *Becn1*^+/-^ mice were infected with 10 *T*. *gondii* ME49 tissues cysts. Beginning at 4 weeks post-infection, mice were treated with or without Saracatinib (10 mg kg^-1^ oral gavage 2x per day; 5 days per week) for 2 weeks. (*A*) Representative images of retinas and brains as well as histopathological scoring. Retina: arrowhead = vitreal inflammation; arrow = perivascular inflammation; asterix = disruption of retinal architecture. Brain: arrowhead = perivascular inflammation; arrow, tissue cyst; asterix; microglial nodule. Scale bar, 50 μm. Data are shown as Data are shown as mean ± SEM from mice pooled from 2 independent experiments: n = 5 (DMSO, Beclin1+/+), 6 (Saracatinib, Beclin1 +/+), 3 (DMSO, Beclin1+/-), and 4 (Saracatinib, Beclin1+/-). Statistical significance was determined using two-way ANOVA with Tukey multiple comparison test. (*B*) *T*. *gondii B1* gene was examined in the eye by qPCR. Levels were compared to those of one control mouse that was given an arbitrary value of 1. Number of *T*. *gondii* tissue cysts were counted in brain homogenates. Bars represent mean ± SEM from mice pooled from 2 independent experiments: n = 5 (DMSO, Beclin1+/+), 6 (Saracatinib, Beclin1 +/+), 3 (DMSO, Beclin1+/-), and 3 or 4 (Saracatinib, Beclin1+/-). Statistical significance determined using one-way ANOVA with Tukey’s multiple comparison test (*p<0.01,***p<0.001, ****p<0.0001).

Finally, we determined if the protective effect of Saracatinib was dependent on the autophagy protein Beclin 1. *Becn 1*^*+/+*^ and *Becn 1*^*+/-*^ mice that had been infected with ME49 tissue cysts for four weeks to develop pre-established disease were then treated with Saracatinib for 2 weeks. Saracatinib-treated *Becn 1*^*+/+*^ mice demonstrated improvement in ocular and cerebral histopathology as well as parasite load (Fig 7A). In contrast, autophagy-deficient *Becn 1*^*+/-*^ mice did not respond to Saracatinib administration and remained with severe histopathology and elevated parasite loads (Fig 7A and 7B). Taken together, Saracatinib triggered accumulation of PTEN around tachyzoites in neural tissue reducing expression of phospho-S473 Akt, triggered marked reduction in parasite load and induced protection against ocular and cerebral toxoplasmosis that were dependent on Beclin 1.

## Discussion

**Fig 8:**
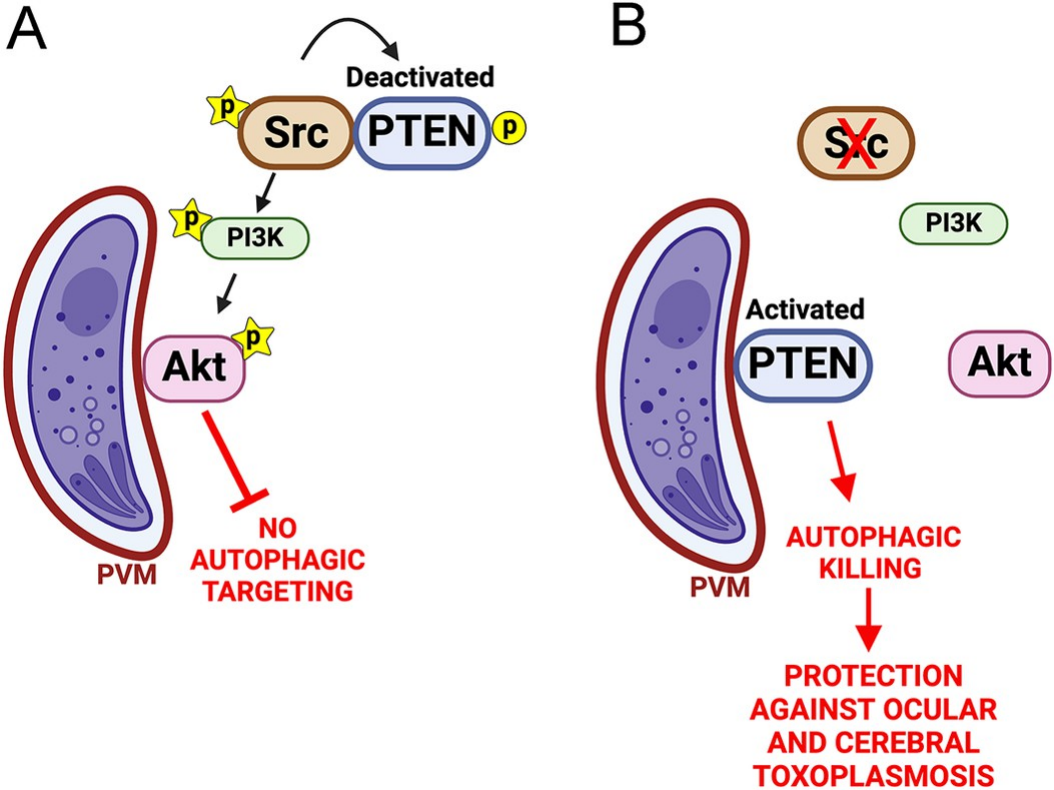
Inhibition of Src signaling induces autophagic targeting of *T*. *gondii* via PTEN-mediated deactivation of Akt. *T*. *gondii* can activate Src independently of EGFR. *(A)* Activated Src associates with PTEN inducing phosphorylation of residues that deactivate PTEN. Src promotes PI3K and Akt activation. Activated Akt is recruited to the PVM and blocks autophagic targeting of the parasite. *(B)* Deficiency of Src or treatment with Saracatinib enables PTEN activation, recruitment of PTEN to the PVM and autophagic killing of *T*. *gondii*. These events occur both in EGFR^+^ and EGFR^-^ cells. ★ represents phosphorylation of residues indicative of activated molecules: phospho-Tyr 416 Src; phospho-Y199 PI3K, phospho-Ser473 Akt. 〇 represents phosphorylation of residues indicative of deactivated PTEN: phospho-Tyr240, phospho-Ser380 and phospho-Thr382/383. Figure generated with BioRender (with permission).

Avoidance of autophagy is key to the survival of *T*. *gondii* since this protozoan is unable to withstand the lysosomal environment. We report that the ubiquitously expressed signaling molecule, Src, is essential for preventing autophagic targeting of *T*. *gondii*, an effect that does not require the presence of EGFR, the host cell molecule previously reported to protect the parasite against autophagy (Fig 8). Knockdown of Src or pharmacologic inhibition of Src using Saracatinib resulted in entrapment of *T*. *gondii* by an LC3^+^ structure, vacuole-lysosomal fusion and killing of the parasite dependent on ULK1 and lysosomal enzymes. Src promoted activation of Akt, a central negative regulator of autophagy. Parasite killing in cells deficient in Src signaling was ablated by expression of CA-Akt, indicating that Src promoted parasite survival through Akt activation. Src regulated the activity of Akt via PTEN. Src associated with PTEN in *T*. *gondii*-infected cells, an effect that was dependent on Src activation (Fig 8). Src promoted an inactive conformation of PTEN and therefore, PTEN was not detected around the PV. However, PTEN acquired an active conformation and migrated around the PV in cells treated with Saracatinib. This was accompanied by marked reduction in the expression of activated Akt in the PVM and autophagic killing of *T*. *gondii*. Treatment of mice with ocular and cerebral toxoplasmosis with Saracatinib led to replacement of activated Akt for PTEN around the tachyzoites, marked reduction in parasite load and histopathology that were dependent on the autophagy protein Beclin 1. In summary, these results uncovered the importance of the Src–PTEN–Akt pathway as a central regulator of *T*. *gondii* survival and support the key role of this pathway in neural toxoplasmosis.

Our studies revealed that activated Akt surrounded intracellular tachyzoites. In this regard, increasing evidence supports that Akt is not only functional at the level of the plasma membrane but may also act in intracellular signaling hubs [49,50] whereby cytoplasmic Akt would be recruited to compartments enriched for phosphatidylinositols leading to allosteric activation of membrane-associated Akt [51]. Activated Akt within *T*. *gondii*-infected cells co-localized with GRA7 supporting that it is associated with the PVM. This finding together with the evidence that activated Akt promotes *T*. *gondii* survival supports that this molecule acts at the level of the PVM to protect the parasite. Such spatial compartmentalization of Akt activation to the PVM would result in a specific effect targeted against this compartment. Of note, Akt in the lysosomal membrane can regulate chaperone-mediated autophagy [52].

PTEN is a major negative regulator of Akt signaling by acting as a phosphatase against phosphatidylinositols [32]. Our studies revealed that Src functions through PTEN to regulate activation of Akt in *T*. *gondii*-infected cells. Src associated with PTEN in *T*. *gondii*-infected cells. This was accompanied by phosphorylation of PTEN in Y240, a residue reported to be directly phosphorylated by Src [37], and by maintenance of S380, T382 and T383 phosphorylation, a hallmark of the inactive conformation of PTEN [33–35]. However, genetic, or pharmacologic inhibition of Src resulted in dephosphorylation Y240, S380, T382 and T383 of PTEN, markers of activated PTEN. Indeed, PTEN was recruited around the PV, and this was accompanied by marked reduction in the accumulation activated Akt. Moreover, PTEN was required for killing of *T*. *gondii* upon inhibition of Src signaling. In addition to promoting the inactive state of PTEN, Src promotes activation of PI3K [53], a molecule that stimulates phosphatidylinositol expression. PI3K has been reported to activate Akt in *T*. *gondii*-infected cells, an event that occurs in both an EGFR-dependent and independent manner [18,54]. Taken together, there are at least two levels at which Src controls the Akt pathway in *T*. *gondii*-infected cells: deactivation of PTEN and activation of PI3K.

*T*. *gondii* activates Src during host cell invasion by triggering integrin-dependent Focal Adhesion Kinase activation [19]. The parasite maintains Src activation during its intracellular state through parasite-induced PKCα/β signaling [20]. While this work uncovered that Src is central for avoidance of autophagic targeting of *T*. *gondii* and parasite survival in cells deficient in EGFR, Src is also a component of the EGFR-dependent strategy utilized by the parasite to avoid killing by autophagy. Src activates EGFR in cells that express this receptor which in turn promotes Akt activation [20]. Here, we show that Src can directly regulate Akt activation in the absence of EGFR. It is likely that both mechanisms of regulation of Akt activity are operative in cells that express EGFR.

Saracatinib penetrates the blood-brain barrier and is well tolerated in animals and humans, including elderly individuals [27]. Our studies indicate that the effects of Saracatinib in *T*. *gondii* infection are mediated by inhibition of Src since the anti-*T*. *gondii* activity of Saracatinib was dependent on Src (not present in Src-deficient cells), and Yes, Fyn as well as c-Abl do not play an appreciable role in regulating survival of *T*. *gondii*. Saracatinib did not exhibit appreciable direct anti-parasitic activity since the replication of *T*. *gondii* was unaffected in cells that remained infected following treatment. Moreover, Saracatinib replicated the effect of Src knockdown further supporting that this drug acts as a Src inhibitor in models of *T*. *gondii* infection.

Our *in vivo* studies indicated that the interplay between Src, PTEN and Akt takes place around tachyzoites in neural tissue. We chose to examine the *in vivo* relevance of this cascade by testing the effects of a Src kinase inhibitor due to the high level of Src expression in neural tissue (approximately 8–10 times than other cells) [40], the ability of Saracatinib to induce potent inhibition of Src signaling [24] and penetrate the blood brain barrier [27]. The fact that Saracatinib induced recruitment of PTEN around tachyzoites, diminished expression of activated Akt and markedly reduced the parasite load in the brain and eye in a Beclin 1-dependent manner supports that the Src-PTEN-Akt dependent regulation of autophagic control of *T*. *gondii* is operative *in vivo* in neural tissue.

Whereas Saracatinib can control an intracellular pathogen *in vivo* (this study and [55]), it should be considered that Src family kinases have been implicated as signaling molecules downstream of receptors in immune cells [4,56]. However, their role is considered to be that of modulators of responses in these cells rather than “on-off switches” [4,56]. This is because these kinases can trigger stimulatory or inhibitory pathways and they can exhibit redundant effects in immune cells [4,56]. Moreover, *in vitro* and *in vivo* studies revealed that administration of low doses of Saracatinib (similar to those used in our work) to previously primed T cells resulted in increased T cell expansion and IFN-γ production following antigenic stimulation [48]. Interestingly, Saracatinib did not inhibit the activity of Lck and Fyn in T cells (Src family kinases involved in TCR signaling) whereas the Src family kinase inhibitor Dasatinib reduced Lck and Fyn kinase activity in T cells and impaired IFN-γ production [48]. Indeed, our studies revealed that Saracatinib caused a modest increase in serum levels of IFN-γ in *T*. *gondii*-infected mice and did not affect serum levels of TNF-α and IL-12 p40. The reduction on IFN-γ, TNF-α, IL-12 p40 and NOS2 mRNA levels in the brain and eye are consistent with the profound decrease in parasite load in these organs.

While we used Saracatinib as a pharmacological tool to probe the role of a host kinase in cerebral and ocular toxoplasmosis, the striking effects of this drug in reducing parasite load and histopathology support that Saracatinib can have therapeutic applications in the treatment of ocular and cerebral toxoplasmosis. Finding that Saracatinib and anti-*T*. *gondii* antibiotics cooperate to control the parasite can result in novel and improved therapeutic regimens based on the administration of Saracatinib at doses even lower than those used in this study plus administration of low doses of antibiotics with the goal to achieve optimal control of toxoplasmosis while minimizing side-effects.

In summary, our studies identified that Src acts as a major determinant of *T*. *gondii* survival by maintaining PTEN in a deactivated state and thus, promoting an intracellular signaling hub consisting of activated Akt associated with the PVM that protects the parasite against autophagic killing. Inhibition of Src results in an activated state of PTEN, recruitment of PTEN around the PV and reduction in the expression of activated Akt. As a result, *T*. *gondii* is no longer protected against autophagic killing. These findings are important because these cascades of events are not restricted by the presence of EGFR (in contrast to strategies of parasite survival identified previously) and are operative in neural tissue. Continued work on the molecular events activated by *T*. *gondii* to manipulate host cell signaling will be central to fully understand the strategies used by the parasite to ensure its survival and rationalize novel approaches for treatment of toxoplasmosis.

## Materials and methods

### Ethics statement

All studies were performed in accordance with regulations of the Guide for the Care and Use of Laboratory Animals and the National Institute of Health. The protocol received approval from the Institutional Animal Care and Use Committee (IACUC) of Case Western Reserve University (Protocol Number: 2015–0130).

### Mice

C57BL/6 mice (Jackson Laboratories, Bar Harbor, ME) or *Becn 1*^+/-^ (C57BL/6 background; gift from Beth Levine, University of Texas Southwestern, Dallas, TX) were bred at the Animal Resource Center at Case Western Reserve University. Female mice between the ages of 6 to 8 weeks old were infected with 10 cysts of the ME49 *T*. *gondii* for four weeks. In certain experiments, mice were euthanized at 4 weeks to evaluate severity of ocular and cerebral toxoplasmosis. Littermates were randomly assigned and treated with vehicle or Saracatinib (10 mg kg^-1^ or 15 mg kg^-1^; SelleckChem, Houston, TX) via oral gavage two times per day, 5 days a week for 4 weeks. Mice were also treated with trimethoprim-sulfamethoxazole (120:600 mg kg^-1^; Dot Scientific Inc, Burton, MI and Sigma, St. Louis, MO). In certain experiments, mice received Dexamethasone (0.1 mg kg^-1^, i.p injection) for 2 weeks followed by Saracatinib treatment and continued administration of Dexamethasone. For immunofluorescence studies, mice were infected with 10 ME49 cysts for 6 weeks before treatment with vehicle or Saracatinib (10 mg kg^-1^) twice daily for 4 days. *Becn 1*^*+/+*^ and *Becn 1*^*+/-*^ mice were infected with 10 ME49 tissue cysts for 4 weeks followed by treatment with Saracatinib (10 mg kg-1) for 2 weeks. All *T*. *gondii* infection experiments were conducted in animal Biosafety Level 2 facilities.

### T. gondii

*T*. *gondii* tachyzoites of the RH and PTG strains were used to infect mammalian cells. RFP- expressing RH *T*. *gondii* (a gift from Boris Striepen, University of Pennsylvania) and GFP- expressing PTG *T*. *gondii* (a gift from George Yap, Rutgers University) were used for this study. Tachyzoites were maintained in human foreskin fibroblasts as described [57].

### Mammalian cells and *in vitro* infection with *T*. *gondii*

Chinese Hamster Ovary (CHO; a gift from Cathleen Carlin, Case Western Reserve University), human retinal pigment epithelial (RPE) cells (ARPE-19; American Type Culture Collection, ATCC, Manassas, VA), and mouse endothelial cells (mHEVc) transduced with Src shRNA or control encoding lentiviral vectors [58] were cultured in complete media. Mouse brain endothelial cells expressing WT or DN EGFR were isolated as described [21]. Endothelial cells were cultured in medium containing Endothelial Cell Growth Supplement (ECGS; Sigma-Aldrich, St Louis, MO). Mammalian cells were infected with tachyzoites of the RH or PTG stains of *T*. *gondii;* RH strain was used unless otherwise indicated. Cells were fixed at 2 hours or 24 hours followed by staining using Hema 3 Manual Staining System (Fisherbrand, Hampton, NH) [21]. At least 100 to 200 cells per monolayer were counted by examining a minimum of 5 random areas per monolayer. The percentage of infected cells, tachyzoites and vacuoles per 100 cells, and tachyzoites per vacuole were counted using light microscopy [21]. Three independent experiments were performed. Saracatinib, Gefitinib (LC Laboratories, Woburn, MA) and lysosomal inhibitors leupeptin and pepstatin (10 μM; Millipore Sigma, Burlington, MA) were used in specified experiments. Each cell monolayer was generated and infected independently.

### Transfection

Cells were transfected with mouse Src siRNA (target sequence: 5’ GGGAGAACCUGGUGUGCAAUU 3’), mouse ULK1 siRNA (Ambion, Carlsbad, CA), mouse PTEN siRNA (target sequence: 5’ GUAUAGAGCGUGCAGAUAAUU3’), or control siRNA (Santa Cruz Biotechnology, Santa Cruz, CA) using TransIT-X2 Dynamic Delivery System (Mirus, Madison, WI).

### Histology

Brains and eyes were fixed in 10% formalin before paraffin embedding. Hematoxylin & eosin (H&E) and periodic acid Schiff hematoxylin (P.A.S.H) were used to stain sections from eyes and brains respectively. Brain and ocular histopathology were scored as described previously [20,21].

### Immunofluorescence

Mammalian cells were infected with RFP-*T*. *gondii* (RH) and fixed with 4% paraformaldehyde. Cells were stained with antibodies against LC3 (MBL International, Woburn, MA), LAMP1 (DSHB, Iowa City, IA), or GRA7 (gift from John Boothroyd, Stanford University, Stanford, CA) followed by a fluorescently conjugated secondary antibodies (Jackson ImmunoResearch Laboratories, West Grove, PA). LC3 and LAMP1 were evaluated for accumulation at 5- and 7 hours post-infection, respectively [18–21,58]. Cells were stained with antibodies against PTEN (Santa Cruz Biotechnology, Dallas, TX) or anti-phospho S473 Akt (Novus, Centennial, CO). Frozen brain sections were stained with antibodies against PTEN (Novus, Centennial, CO), phospho S473 Akt, SAG-1 (Invitrogen, Waltham, MA) or *T*. *gondii* (Biogenex, Fremont, CA). Parasites were evaluated blindly in multiple random fields of view and 100 parasites were examined per group. In-out assays were performed as described using RFP-*T gondii*, anti-SAG-1 antibody (Invitrogen, Waltham, MA) and an Alexa Fluor 488-conjugated secondary antibody. Olympus FV1200 confocal microscope (Evident/Olympus, Waltham, MA) equipped with UPlanSApa 60x and 100x 1.45NA objectives was used to evaluate the slides [59,60]. Deconvolved images were generated with Huygens Essential 23.10 software (Scientific Volume Imaging, Hilversum, the Netherlands) using the Standard deconvolution profile in Deconvolution Express. Images were processed in Photoshop CC 19.1.1. using similar linear adjustments for all samples.

### Immunoblot

Membranes were probed with antibodies against Src, phospho-Y416 Src, Akt, phospho-S473 Akt, PTEN, phospho-S380/T382/T383 PTEN, PI3K, phospho-Y458/Y199 PI3K, ULK1, EGFR, HA tag (all from Cell Signaling, Danvers, MA), phospho-Y240 PTEN (Sigma) and Actin (Santa Cruz Biotechnologies) followed by incubation with secondary antibodies (Cell Signaling and Santa Cruz Biotechnologies). Three independent experiments were performed.

### Real-time quantitative PCR

Genomic DNA was isolated using a DNeasy isolation kit (Qiagen, Germantown, MD) which was used as the template to measure *T*. *gondii B1* gene expression. Samples were normalized to *L32*. RNA was isolated then reverse transcribed using a RNeasy Plus Kit and a Quantitect Reverse Transcription kit (Qiagen), respectively. cDNA was used as a template to measure IFN-γ, IL-12 p40, TNF-α, and NOS2 which were normalized to 18S rRNA [20]. In addition, cDNA was used to measure EGFR. Primers for EGFR were generated using the NCBI Primer-Blast (FWD: 5’ AACAGAGCCCCGAATGACTG 3’, REV: 5’ CGTTTTCGGACAATGTGGCG 3’). SYBR Green master mix was used to evaluate gene expression. Samples were run using StepOne Real-Time PCR system (Applied Biosystems, Waltham, MA). Relative gene expression was calculated using the ΔΔCt method. Two to three technical replicates were run per sample and data shown represent the average of each sample.

### ELISA

IFN-γ and IL-12 p40 were measured using ELISA kits from R&D (Minneapolis, MN). TNF-α was measured using a kit from eBioscience (San Diego, CA). Anti-*T*. *gondii* IgG was detected by ELISA [20].

### Statistics

Experimental results were analyzed for statistical significance using a two-tailed, unpaired Student’s *t-test*, one-way ANOVA, or two-way ANOVA with Tukey’s or Dunnett’s multiple comparison’s test (GraphPad Prism). Tukey’s multiple comparisons test was used to evaluate the presence of significant differences among all groups, while Dunnett’s multiple comparisons test was used to evaluate the presence of significant differences between experimental groups and the control. Means and standard error were computed. Differences were considered statistically significant if p<0.05.

## Supporting information

S1 Fig Assessment of potential cell toxicity of Saracatinib. CHO cells and retinal pigment epithelial cells (RPE) were treated with various concentrations of Saracatinib (1 nM– 1000 μM) followed by MTT assay 24 hrs. after incubation with Saracatinib. The data are pooled from duplicate or triplicate independent samples obtained in 3 different experiments. Data points are demonstrated as circles, triangles, or squares corresponding to replicates within each experiment. Data are shown as mean ± SEM. Significance was determined by comparing all groups to the control using one-way ANOVA with Holm-Sidak multiple comparison test (*p<0.05).
(PDF)

S2 Fig Saracatinib does not affect invasion of mammalian cells or intracellular replication of the parasite. (A) CHO cells were treated with Saracatinib (1 μM) and infected with RH-RFP *T*. *gondii* for 2 hours. Cells were fixed with Paraformaldehyde (PFA) and were not permeabilized prior to staining with a tachyzoite marker, SAG1 (green). Intracellular cells will express red fluorescence while extracellular parasites stain for SAG1 and appear green or yellow in color. Data are shown as mean ± SEM. The graphs show data from 7 independent monolayers pooled from 2 different experiments. (B) CHO cells were treated with Saracatinib (1 μM) and infected with RH-RFP *T*. *gondii* for 24 hours. Number of tachyzoites per vacuole were determined by light microscopy and quantified as percentage of total vacuoles. Data are shown as mean ± SEM. The graphs show data from 6 independent monolayers pooled from 3 different experiments. No significant difference was found between control and Saracatinib-treated groups following statistical analysis using two-way, unpaired Student’s t test.
(PDF)

S3 Fig Src but not Fyn, Yes or c-Abl regulate *T*. *gondii* survival. (A) Wildtype fibroblasts (MEF), MEFs deficient in Src/Yes/Fyn (SYF), or SYF MEFs reconstituted to express Src were infected with RH *T*. *gondii*. Percentage of infected cells and number of tachyzoites per 100 cells were determined by light microscopy. Data shown as mean ± SEM. The graphs show data from 3 independent monolayers per group pooled from 3 different experiments. Significance for the percentages of infected cells was determined using two-way ANOVA with Tukey’s multiple comparison test. Statistical analysis shown represents comparisons of 2 v. 24 hrs. Significance for tachyzoites per 100 cells was determined using one-way ANOVA with Dunnett’s multiple comparison test comparing groups to wild-type (WT) control. (*p<0.05, ****p<0.0001). (B) CHO cells were treated with Imatinib, a c-Abl inhibitor (0.3 μM or 1μM) and infected with RH-RFP *T*. *gondii* for 2 or 24 hours. Data are shown as mean ± SEM from four independent monolayers per group pooled from 2 different independent experiments. No significant difference was found comparing all groups using one- or two-way ANOVA as described above.
(PDF)

S4 Fig Endothelial cells deficient in Src exhibit toxoplasmacidal activity that is regulated by ULK1 and Akt. (A) Mouse endothelial cells (mHEVc) transduced with control shRNA or Src shRNA were transfected with control siRNA or ULK1 siRNA followed by infection with RH RFP *T*. *gondii*. Data shown as mean ± SEM. The graphs show data from 4 independent monolayers pooled from 2 different experiments. (B) mHEVc cells transduced with control shRNA or Src shRNA were transfected with a plasmid that encodes WT-Akt or CA-Akt followed by challenge with *T*. *gondii*. Monolayers were examined as above. All data are shown as mean ± SEM. The graphs show data from 4 independent monolayers pooled from 2 different experiments. All significance was determined using two-way ANOVA with Dunnett’s multiple comparison test comparing Ctr vs. Src shRNA in each condition. (*p<0.05, **p<0.01, ***p<0.001, ****p<0.0001).
(PDF)

S5 Fig Tissue from B6 Mice express endogenous levels of Src protein expression. Western blot analysis of Src expression from brain, retina, lung, liver and spleen of healthy two B6 mice. 50 ug of each tissue lysate were loaded and probed for Src and Actin. Relative density of Src was normalized to total Actin and compared to the Brain Sample #1. Densitometry analysis of blots shown from two mice.
(PDF)

S6 Fig B6 mice develop cerebral toxoplasmosis at 4-weeks post-infection. B6 female mice aged 6–8 weeks-old were infected with 10 ME49 *T*. *gondii* via i.p. injection. Following 4 weeks of infection, mice were euthanized and assessed for parasite load in the brain by counting the number of tissue cysts found per brain. Data are shown as mean ± SEM from 10 mice pooled from 2 independent experiments.
(PDF)

S7 Fig Saracatinib treatment provides protection against ocular and cerebral toxoplasmosis in Dexamethasone-treated mice. Female B6 mice aged 6–8 weeks were infected with 10 ME49 tissue cysts for 3 weeks. Mice were then treated with Dexamethasone (0.1 mg kg-1, i.p injection) for 10–14 days before treatment with Saracatinib (10 mg kg-1 oral gavage 2x day, 5 days a week) for 2 weeks. (A-B) Representative images of retinas and brains treated with PBS or Dexamethasone as well as histopathological scoring. Retina: arrowhead = vitreal inflammation; arrow = perivascular inflammation; asterisk = disruption of retinal architecture. Brain: arrowhead = perivascular inflammation; arrow, tissue cyst; asterisk; microglial nodule. Scale bar, 50 μm. Data represents mean ± SEM from 8 mice per group pooled from 2 independent experiments. Statistical significance was determined using unpaired Student’s t test comparing. (*p<0.05, **p<0.01, ***p<0.001). (C-D) Representative images of retinas and brains treated with Dexamethasone with or without Saracatinib treatment as well as histopathological scoring. Retina: arrowhead = vitreal inflammation; arrow = perivascular inflammation; asterisk = disruption of retinal architecture. Brain: arrowhead = perivascular inflammation; arrow, tissue cyst; asterisk; microglial nodule. Scale bar, 50 μm. Data are shown as mean ± SEM from mice pooled from 2 independent experiments: Retina- n = 13 (Dexamethasone) and 9 (Dexamethasone + Saracatinib). Brain- n = 8 (Dexamethasone) and 10 (Dexamethasone + Saracatinib). Statistical significance was determined using unpaired Student’s t test (***p<0.001, ****p<0.0001). (E) *T*. *gondii* B1 gene was examined in the eye using qPCR. Levels were compared to those of one control mouse that was given an arbitrary value of 1. Bars represent mean ± SEM pooled from mice pooled from 2 independent experiments: n = 5 (PBS), 7 (Dexamethasone), and 6 (Dexamethasone + Saracatinib). Statistical significance was determined one-way ANOVA with Tukey’s multiple comparison test (**p<0.01). (F) Number of *T*. *gondii* tissue cysts were counted per brain homogenate. Data are shown as mean ± SEM from mice pooled from 2 independent experiments: n = 4 (PBS), 12 (Dexamethasone) and 10 (Dexamethasone + Saracatinib). Statistical significance was determined using one-way ANOVA with Tukey’s multiple comparison test (***p<0.001).
(PDF)

S1 Data Numerical data corresponding to main figures and supplemental figures presented in the study. Each table lists the individual data points for presented biological replicates.
(PDF)

S1 File Replicate western blot images used for densitometry from Figs 1, 2, 4 and 5. (PDF)
