# Supplementary figures and images for: Inhibition of Src signaling induces autophagic killing of Toxoplasma gondii via PTEN-mediated deactivation of Akt
Source: PLoS Pathog. 2025 Jan 27;21(1):e1012907. doi: 10.1371/journal.ppat.1012907 (PMC11801697; doi:10.1371/journal.ppat.1012907)

Supplementary Figure 1

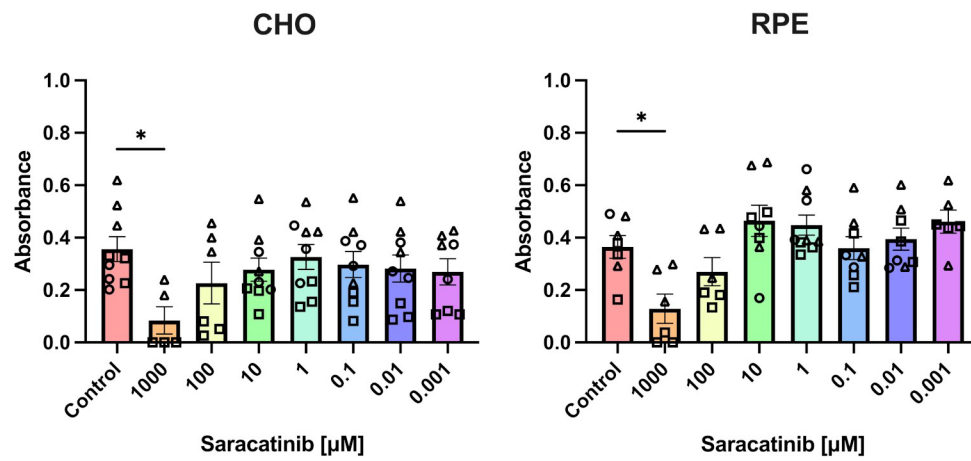

Supplement: S1 Fig — CHO cells and retinal pigment epithelial cells (RPE) were treated with various concentrations of Saracatinib (1 nM– 1000 μM) followed by MTT assay 24 hrs. after incubation with Saracatinib. The data are pooled from duplicate or triplicate independent samples obtained in 3 different experiments. Data points are demonstrated as circles, triangles, or squares corresponding to replicates within each experiment. Data are shown as mean ± SEM. Significance was determined by comparing all groups to the control using one-way ANOVA with Holm-Sidak multiple comparison test (*p<0.05). (PDF) [file ppat.1012907.s001.pdf]

Supplementary Figure 2

A

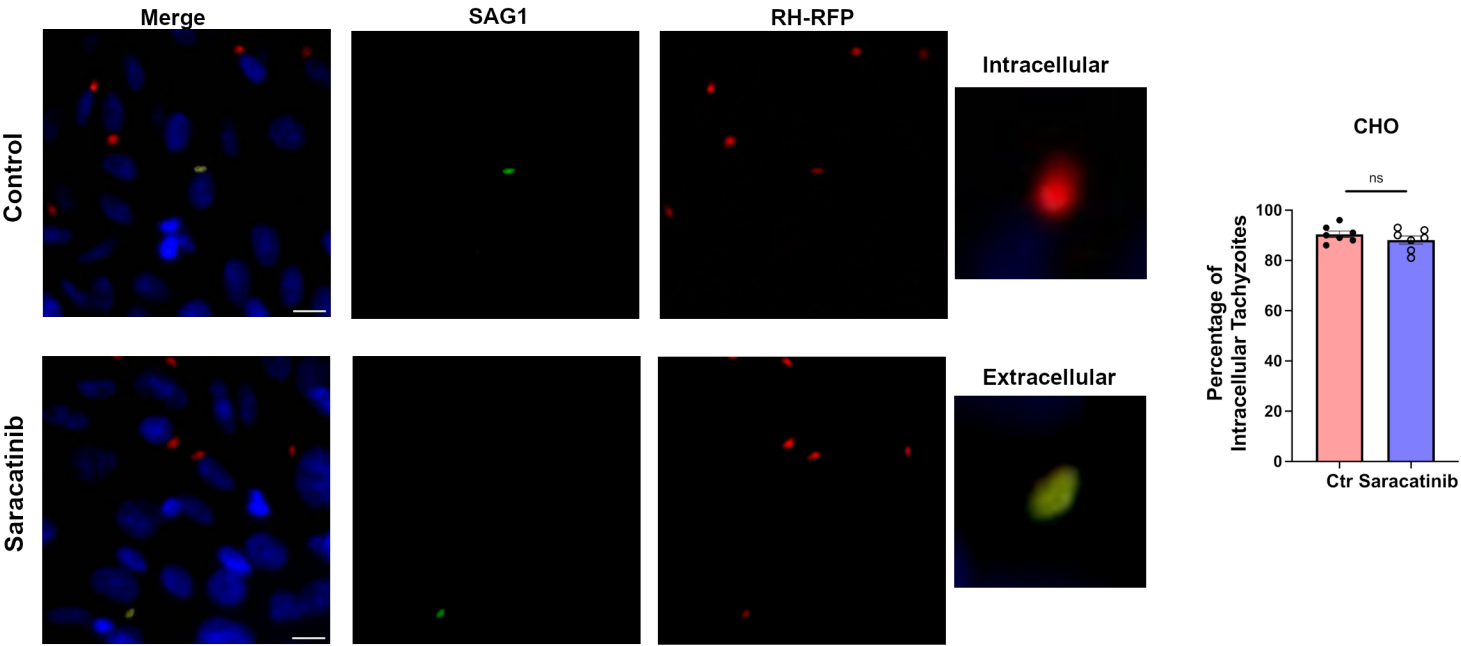

B

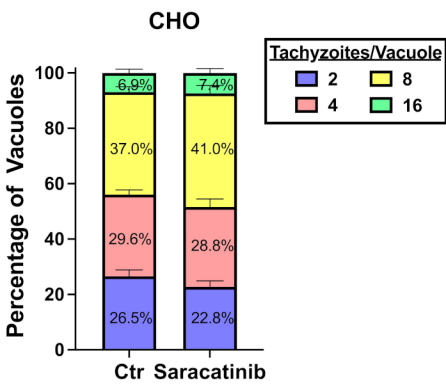

Supplement: S2 Fig — (A) CHO cells were treated with Saracatinib (1 μM) and infected with RH-RFP T. gondii for 2 hours. Cells were fixed with Paraformaldehyde (PFA) and were not permeabilized prior to staining with a tachyzoite marker, SAG1 (green). Intracellular cells will express red fluorescence while extracellular parasites stain for SAG1 and appear green or yellow in color. Data are shown as mean ± SEM. The graphs show data from 7 independent monolayers pooled from 2 different experiments. (B) CHO cells were treated with Saracatinib (1 μM) and infected with RH-RFP T. gondii for 24 hours. Number of tachyzoites per vacuole were determined by light microscopy and quantified as percentage of total vacuoles. Data are shown as mean ± SEM. The graphs show data from 6 independent monolayers pooled from 3 different experiments. No significant difference was found between control and Saracatinib-treated groups following statistical analysis using two-way, unpaired Student’s t test. (PDF) [file ppat.1012907.s002.pdf]

## Supplementary Figure 3

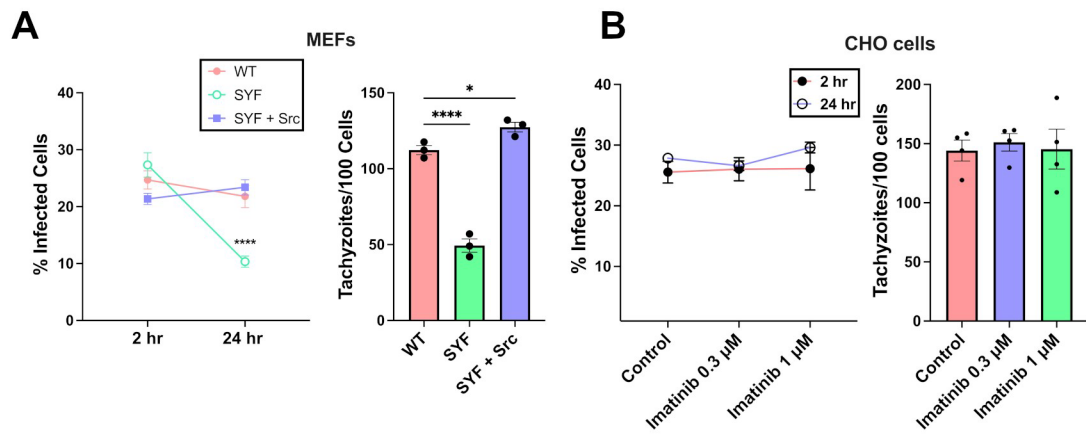

Supplement: S3 Fig — (A) Wildtype fibroblasts (MEF), MEFs deficient in Src/Yes/Fyn (SYF), or SYF MEFs reconstituted to express Src were infected with RH T. gondii. Percentage of infected cells and number of tachyzoites per 100 cells were determined by light microscopy. Data shown as mean ± SEM. The graphs show data from 3 independent monolayers per group pooled from 3 different experiments. Significance for the percentages of infected cells was determined using two-way ANOVA with Tukey’s multiple comparison test. Statistical analysis shown represents comparisons of 2 v. 24 hrs. Significance for tachyzoites per 100 cells was determined using one-way ANOVA with Dunnett’s multiple comparison test comparing groups to wild-type (WT) control. (*p<0.05, ****p<0.0001). (B) CHO cells were treated with Imatinib, a c-Abl inhibitor (0.3 μM or 1μM) and infected with RH-RFP T. gondii for 2 or 24 hours. Data are shown as mean ± SEM from four independent monolayers per group pooled from 2 different independent experiments. No significant difference was found comparing all groups using one- or two-way ANOVA as described above. (PDF) [file ppat.1012907.s003.pdf]

Supplementary Figure 4

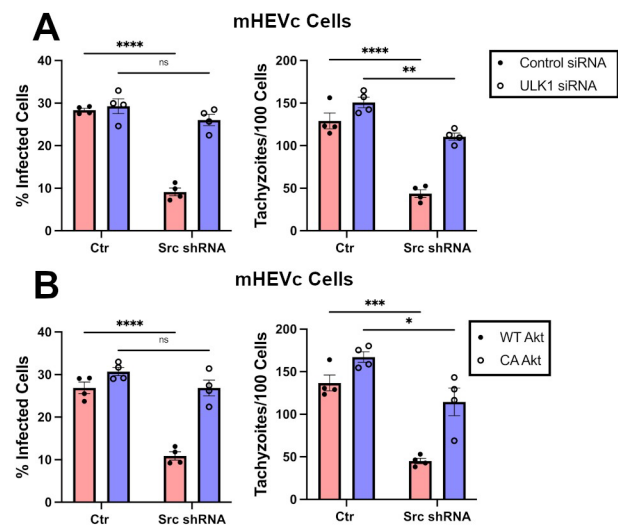

Supplement: S4 Fig — (A) Mouse endothelial cells (mHEVc) transduced with control shRNA or Src shRNA were transfected with control siRNA or ULK1 siRNA followed by infection with RH RFP T. gondii. Data shown as mean ± SEM. The graphs show data from 4 independent monolayers pooled from 2 different experiments. (B) mHEVc cells transduced with control shRNA or Src shRNA were transfected with a plasmid that encodes WT-Akt or CA-Akt followed by challenge with T. gondii. Monolayers were examined as above. All data are shown as mean ± SEM. The graphs show data from 4 independent monolayers pooled from 2 different experiments. All significance was determined using two-way ANOVA with Dunnett’s multiple comparison test comparing Ctr vs. Src shRNA in each condition. (*p<0.05, **p<0.01, ***p<0.001, ****p<0.0001). (PDF) [file ppat.1012907.s004.pdf]

Supplementary Figure 5

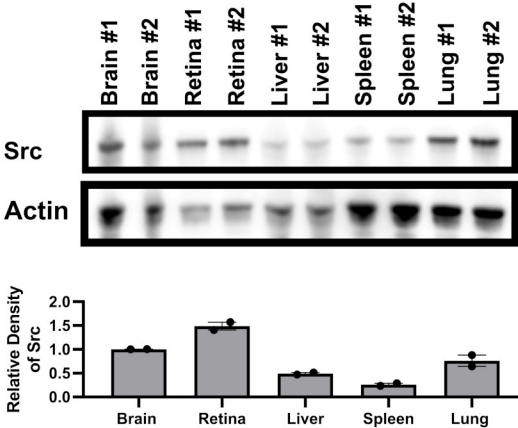

Supplement: S5 Fig — Western blot analysis of Src expression from brain, retina, lung, liver and spleen of healthy two B6 mice. 50 ug of each tissue lysate were loaded and probed for Src and Actin. Relative density of Src was normalized to total Actin and compared to the Brain Sample #1. Densitometry analysis of blots shown from two mice. (PDF) [file ppat.1012907.s005.pdf]

Supplementary Figure 6

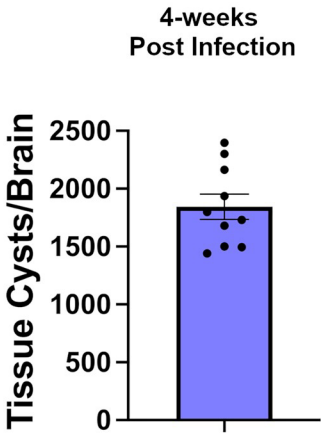

Supplement: S6 Fig — B6 female mice aged 6–8 weeks-old were infected with 10 ME49 T. gondii via i.p. injection. Following 4 weeks of infection, mice were euthanized and assessed for parasite load in the brain by counting the number of tissue cysts found per brain. Data are shown as mean ± SEM from 10 mice pooled from 2 independent experiments. (PDF) [file ppat.1012907.s006.pdf]

# Supplementary Figure 7

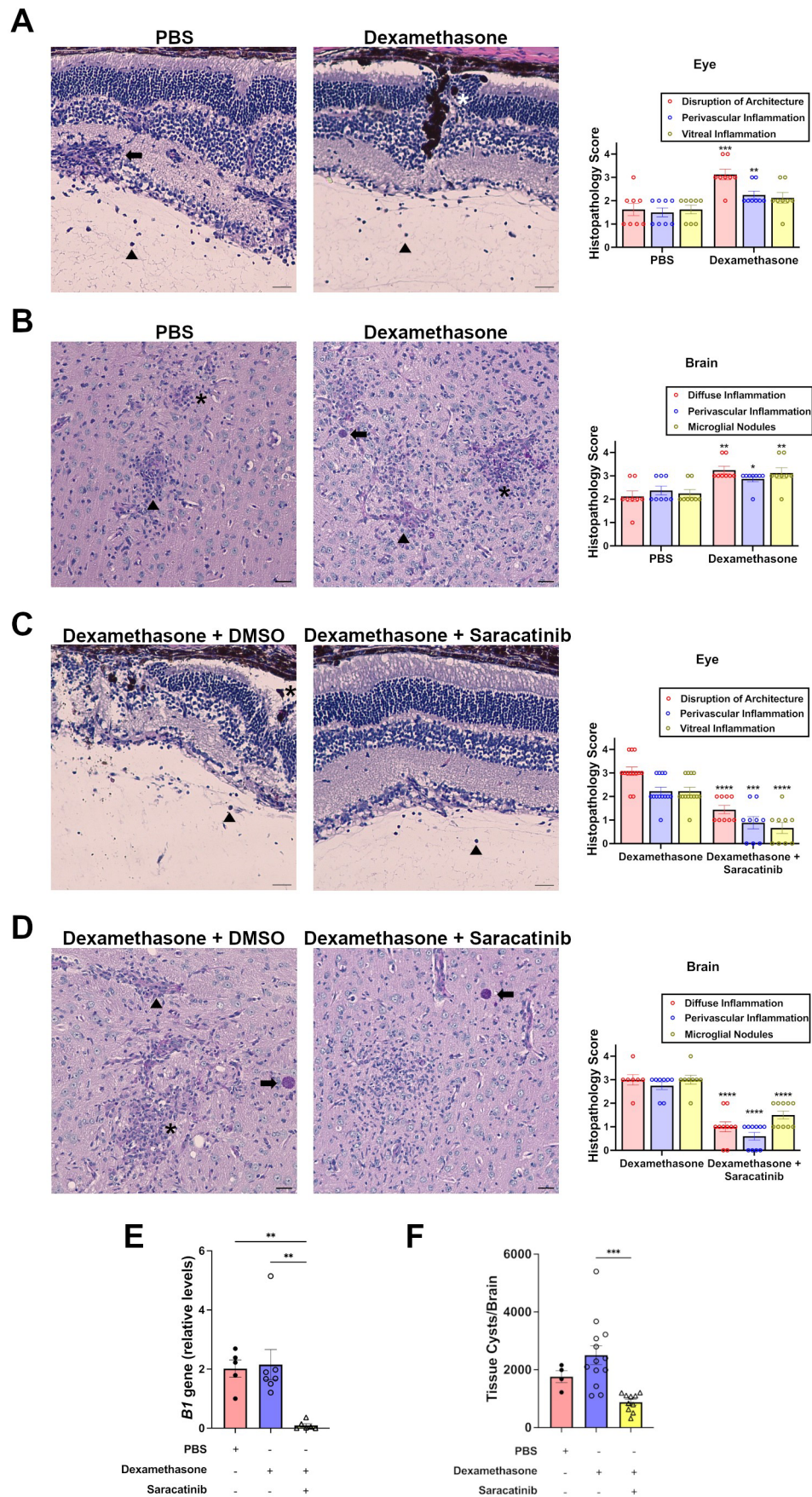

Figure 1A

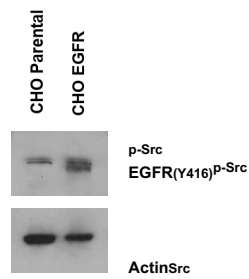

Figure 1B

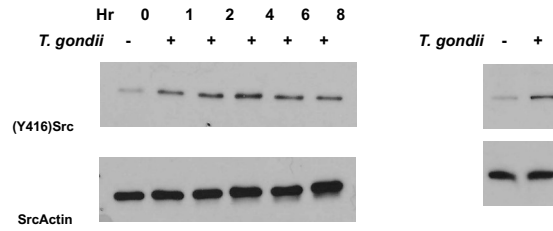

Figure 1C

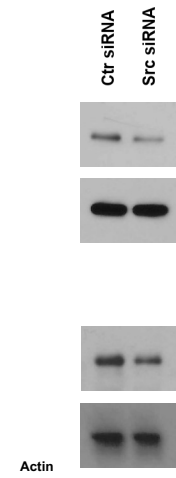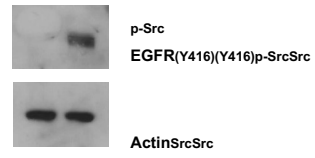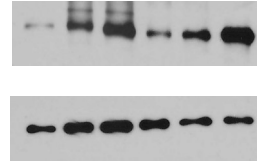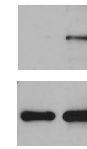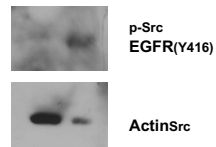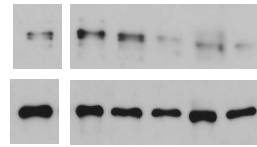

Figure 2A

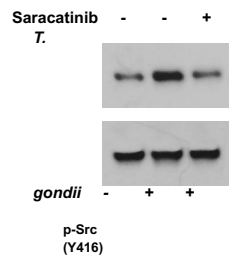

Supplement: S7 Fig — Female B6 mice aged 6–8 weeks were infected with 10 ME49 tissue cysts for 3 weeks. Mice were then treated with Dexamethasone (0.1 mg kg-1, i.p injection) for 10–14 days before treatment with Saracatinib (10 mg kg-1 oral gavage 2x day, 5 days a week) for 2 weeks. (A-B) Representative images of retinas and brains treated with PBS or Dexamethasone as well as histopathological scoring. Retina: arrowhead = vitreal inflammation; arrow = perivascular inflammation; asterisk = disruption of retinal architecture. Brain: arrowhead = perivascular inflammation; arrow, tissue cyst; asterisk; microglial nodule. Scale bar, 50 μm. Data represents mean ± SEM from 8 mice per group pooled from 2 independent experiments. Statistical significance was determined using unpaired Student’s t test comparing. (*p<0.05, **p<0.01, ***p<0.001). (C-D) Representative images of retinas and brains treated with Dexamethasone with or without Saracatinib treatment as well as histopathological scoring. Retina: arrowhead = vitreal inflammation; arrow = perivascular inflammation; asterisk = disruption of retinal architecture. Brain: arrowhead = perivascular inflammation; arrow, tissue cyst; asterisk; microglial nodule. Scale bar, 50 μm. Data are shown as mean ± SEM from mice pooled from 2 independent experiments: Retina- n = 13 (Dexamethasone) and 9 (Dexamethasone + Saracatinib). Brain- n = 8 (Dexamethasone) and 10 (Dexamethasone + Saracatinib). Statistical significance was determined using unpaired Student’s t test (***p<0.001, ****p<0.0001). (E) T. gondii B1 gene was examined in the eye using qPCR. Levels were compared to those of one control mouse that was given an arbitrary value of 1. Bars represent mean ± SEM pooled from mice pooled from 2 independent experiments: n = 5 (PBS), 7 (Dexamethasone), and 6 (Dexamethasone + Saracatinib). Statistical significance was determined one-way ANOVA with Tukey’s multiple comparison test (**p<0.01). (F) Number of T. gondii tissue cysts were counted per brain homogen [file ppat.1012907.s007.pdf]

Figure 1A

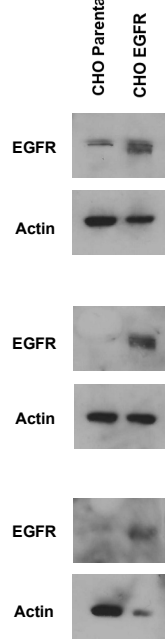

Figure 1B

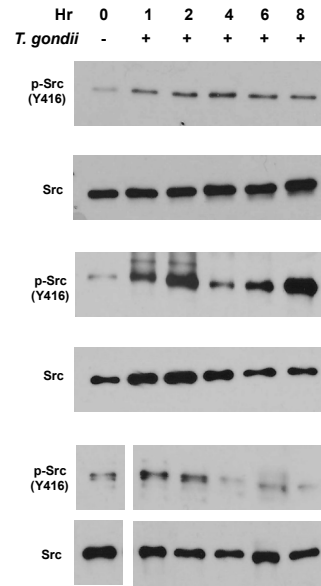

Figure 1C

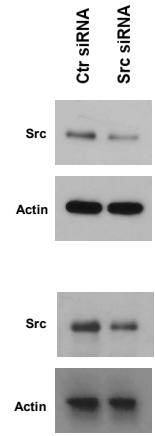

Figure 2A

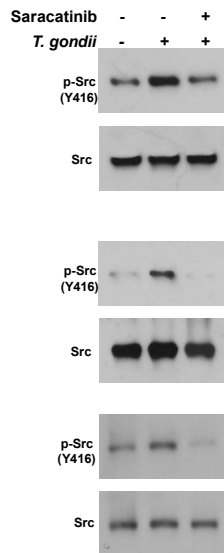

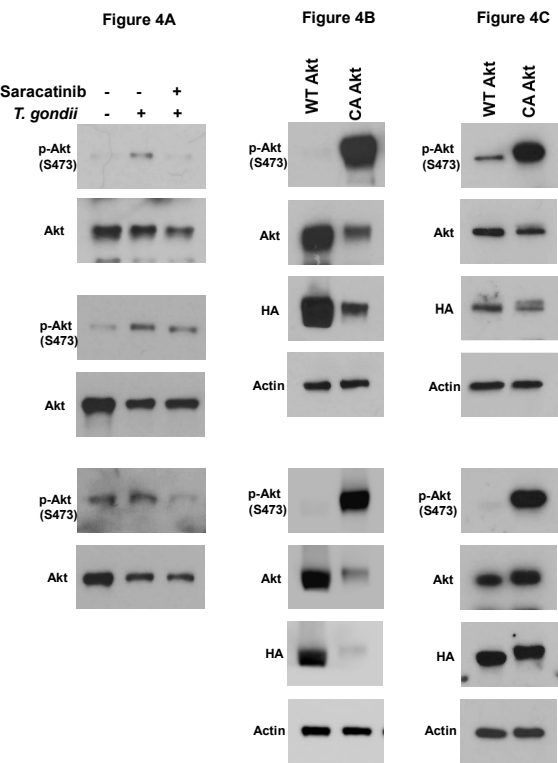

Figure 5A

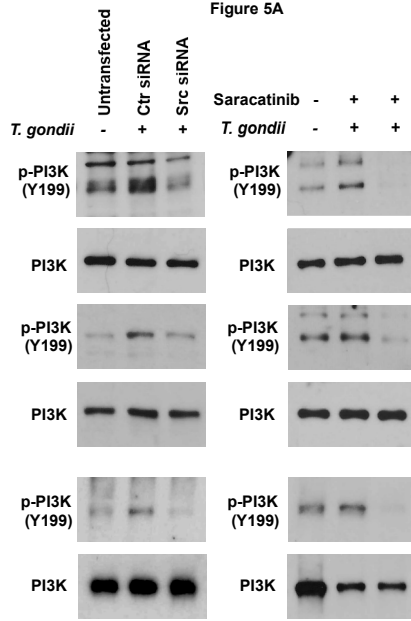

Figure 5D

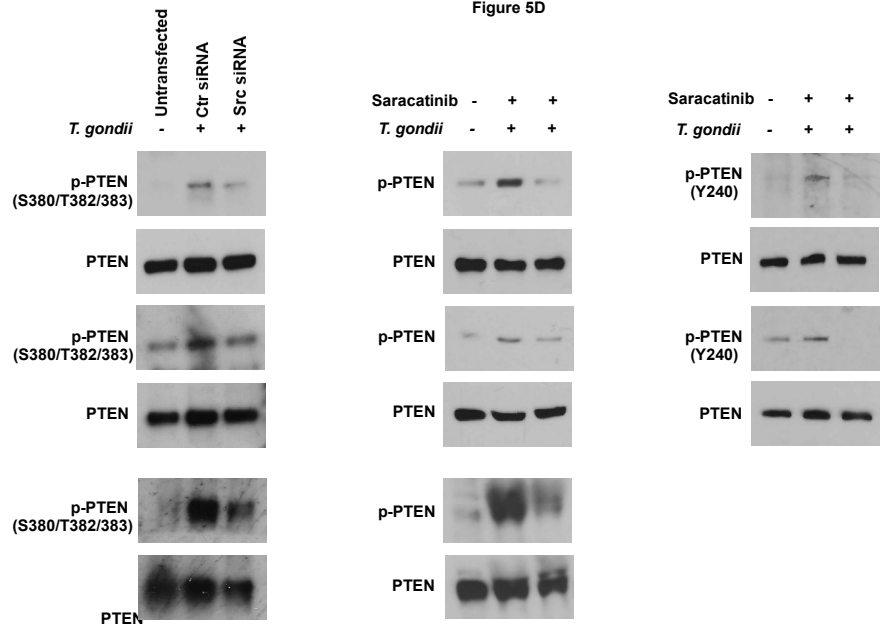

Figure 5E

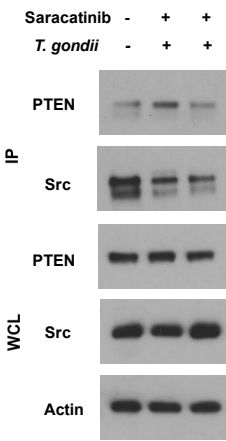

Figure 5F

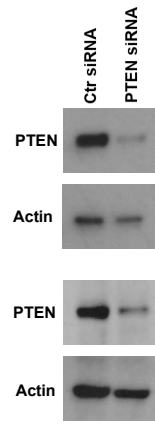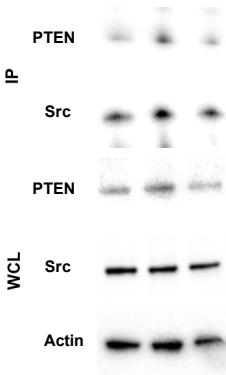

Supplement: S1 File — (PDF) [file ppat.1012907.s009.pdf]
