# Supplementary material for: Inhibition of Src signaling induces autophagic killing of Toxoplasma gondii via PTEN-mediated deactivation of Akt
Source: PLoS Pathog. 2025 Jan 27;21(1):e1012907. doi: 10.1371/journal.ppat.1012907 (PMC11801697; doi:10.1371/journal.ppat.1012907)
Supplement: S1 Data — Each table lists the individual data points for presented biological replicates. (PDF) [file ppat.1012907.s008.pdf]

Figure 1A

Relative Expression Values for:	CHO Parental			CHO EGFR+		
	Replicate 1	Replicate 2	Replicate 3	Replicate 1	Replicate 2	Replicate 3
EGFR (Relative to Actin)	0.829818	1	0.8882617	26.06484	47.55939	83.94408

Densitometry Values for Western	CHO Parental			CHO EGFR+		
	Replicate 1	Replicate 2	Replicate 3	Replicate 1	Replicate 2	Replicate 3
EGFR Expression (Compared to Actin)	1	1	1	2.39	8.744	7.503

Fig 1B

Densitometry Values for Western (CHO)	p-Src (Compared to Src)		
	Replicate 1	Replicate 2	Replicate 3
0	1	1	1
1	1.84	5.08	2.66
2	2.53	7.44	2.72
4	2.43	2.1	0.75
6	1.96	5.98	1.78
8	1.218		0.36

Densitometry Values for Western (DN E	p-Src (Compared to Src)	
	Replicate 1	Replicate 2
0	1	1
2	2.69	7.55

Fig 1C

Densitometry Values for Western (CHO)	Src (Compared to Actin)	
	Replicate 1	Replicate 2
Ctr siRNA	1	1
Src siRNA	0.49025	0.584696

Percentage of Infected Cells (CHO)	2 hr					
	Replicate 1	Replicate 2	Replicate 3	Replicate 4	Replicate 5	Replicate 6
Ctr siRNA	27.6923	26.78571	30.263158	30	25.98425	28.57143
Src siRNA	29.85075	29.5082	28.915663	27.77778	23.40426	24.7191
	24 hr					
	Replicate 1	Replicate 2	Replicate 3	Replicate 4	Replicate 5	Replicate 6
Ctr siRNA	29.5	28.57143	22	21.57676	22.72727	26.5625
Src siRNA	6.467662	5.940594	11.636364	9.920635	10.67961	12.75168

Tachyzoites per 100 Cells (CHO)	24 hr					
	Replicate 1	Replicate 2	Replicate 3	Replicate 4	Replicate 5	Replicate 6
Ctr siRNA	163	167.86	114.4	130.29	125.75	91.406
Src siRNA	36.32	32.67	46.18	45.634	27.18	30.87

Vacuoles per 100 Cells (CHO)	24 hr					
	Replicate 1	Replicate 2	Replicate 3	Replicate 4	Replicate 5	Replicate 6
Ctr siRNA	24.2	23.73444	32.45	31.42857	37.87	32.03
Src siRNA	7.114428	6.534653	12.8	10.9127	11.65	14.76

Tachyzoites per Vacuole (CHO)	24 hr					
	Replicate 1	Replicate 2	Replicate 3	Replicate 4	Replicate 5	Replicate 6
Ctr siRNA	4.727273	5.48951	5.0231125	5.340909	3.32	2.85
Src siRNA	3.607955	4.181818	5.1048951	5	2.33	2.09

Fig 1D

Percentage of Infected Cells (WT/DN EGFR Brain Endothelial	24 hr					
	Replicate 1	Replicate 2	Replicate 3	Replicate 4	Replicate 5	Replicate 6
Ctr siRNA (WT)	32.83582	36.5	34.28	26	36.25	34.065
Src siRNA (WT)	13.54	11.36	11.36	10	17.95	16.88
Ctr siRNA (DN)	28.57	29.661	27.27	28.6	38.805	42.622
Src siRNA (DN)	13.88	13.01	8.61	10.65	18.75	15.51

Tachyzoites per 100 Cells (WT/DN EGFR Brain Endothelial Ce	24 hr					
	Replicate 1	Replicate 2	Replicate 3	Replicate 4	Replicate 5	Replicate 6
Ctr siRNA (WT)	171.6418	223.8095	207.5	165.93	116.19	110
Src siRNA (WT)	51.04	50	55.128	79.2207	44.69	41.11
Ctr siRNA (DN)	202.521	236.4407	242.623	305.97	117.8	138.18
Src siRNA (DN)	67.45	99.07	103.125	96.55	51.67	48.79

Vacuoles per 100 Cells (WT/DN EGFR Brain Endothelial Cells	24 hr					
	Replicate 1	Replicate 2	Replicate 3	Replicate 4	Replicate 5	Replicate 6
Ctr siRNA (WT)	46.26866	47.61905	43.75	40.65	38.095	29
Src siRNA (WT)	16.66667	13.63636	21.79	19.48	12.12	11.11
Ctr siRNA (DN)	47.05882	38.13559	46.268	50.819	29.92	31.36
Src siRNA (DN)	14.81481	15.38462	21.875	18.965	11.68	9.56

Figure 2A

Densitometry Values for Western	p-Src (Compared to Src)		
	Replicate 1	Replicate 2	Replicate 3
Uninfected	1	1	1
Infected DMSO Treated	2.19	3.96	1.43
Saracatinib DMSO Treated	1.04	0.22	0.42

Figure 2B

Percentage of Infected Cells (CHO EGFR+)	24 hr					
	Replicate 1	Replicate 2	Replicate 3	Replicate 4	Replicate 5	Replicate 6
Control	38.5	37	40.5	41.2	40	43
Saracatinib 0.1 uM	16	15	14.1	15.1	17	18
Saracatinib 1 uM	10	9	11.1	10.4	10	12
Saracatinib 10 uM	10	9	9.1	10.4	12	11
Gefitinib 0.1 uM	27	25	26.1	25	29	30
Gefitinib 1 uM	20.5	19	19.2	21.1	23	21
Gefitinib 10 uM	19	18	18.3	19.4	21	22

Tachyzoites per 100 Cells (CHO EGFR+)	24 hr					
	Replicate 1	Replicate 2	Replicate 3	Replicate 4	Replicate 5	Replicate 6
Control	231	226	247	243	236	258
Saracatinib 0.1 uM	94	91	84	89	103	108
Saracatinib 1 uM	59	56	65	61	59	71
Saracatinib 10 uM	57	57	53	59	70	66
Gefitinib 0.1 uM	163	155	155	151	170	177
Gefitinib 1 uM	123	118	113	127	131	137
Gefitinib 10 uM	112	113	107	115	125	129

Figure 2C

Percentage of Infected Cells (CHO- RH stra	2 hr					
	Replicate 1	Replicate 2	Replicate 3	Replicate 4	Replicate 5	Replicate 6
Control	40	35.71	27.69	26.78	23.77	22.22
Saracatinib	38.46	35.714	29.85	29.5	17.47	17.07
	24 hr					
	Replicate 1	Replicate 2	Replicate 3	Replicate 4	Replicate 5	Replicate 6
Control	41.667	38.709	42.622	38.7096	33.018	28.75
Saracatinib	7.865	7.246	7.865169	7.246377	9.83	15.83

Tachyzoites per 100 Cells (CHO- RH strain)	24 hr					
	Replicate 1	Replicate 2	Replicate 3	Replicate 4	Replicate 5	Replicate 6
Control	281.6667	262.903	277.0492	264.5161	183.0188	180
Saracatinib	56.179	46.3768	89.88	46.376	45.08	77.5

Vacuoles per 100 Cells (CHO- RH strain)	24 hr					
	Replicate 1	Replicate 2	Replicate 3	Replicate 4	Replicate 5	Replicate 6
Control	45.8333333	42.5806452	47.54	41.93	39.622	38.75
Saracatinib	8.65168539	7.97101449	8.98	8.69	13.11	20

Tachyzoites per Vacuole (CHO- RH strain)	24 hr					
	Replicate 1	Replicate 2	Replicate 3	Replicate 4	Replicate 5	Replicate 6
Control	6.14545455	6.17424242	5.82	6.3	4.61	4.64
Saracatinib	6.49350649	5.81818182	10	5.33	3.43	3.875

Figure 2D

Percentage of Infected Cells (CHO- PTG strain)	24 hr					
	Replicate 1	Replicate 2	Replicate 3	Replicate 4	Replicate 5	Replicate 6
Control	26.7	32.25	32.06	29.62	24.16	24.698
Saracatinib	13.0081	11.33	7.63	10.7784	8.9	10.55

Tachyzoites per 100 Cells (CHO- PTG strain)	24 hr					
	Replicate 1	Replicate 2	Replicate 3	Replicate 4	Replicate 5	Replicate 6
Control	80	108.0645	86.259	89.81	155.705	146.98
Saracatinib	22.76	29.33	15.9722	21.5568	41.88	37.22

Figure 2E

Percentage of Infected Cells (WT/DN EGFR Bra	24 hr							
	Replicate 1	Replicate 2	Replicate 3	Replicate 4	Replicate 5	Replicate 6	Replicate 7	Replicate 8
Control (WT)	30.1075	27.2727	31.8181	32.6086	23.52	26.86	31.25	32.2
Saracatinib (WT)	12.222	8.247	10.1265	12.1212	15.9	15.2	19.23	14.7
Control (DN)	29.787	31.25	30.693	35.135	30.68	30.76	36	38.09
Saracatinib (DN)	9.0909	8.8888	7.407	8.4905	18.07	20.25	11.29	18.8

Tachyzoites per 100 Cells (WT/DN EGFR Brain	24 hr							
	Replicate 1	Replicate 2	Replicate 3	Replicate 4	Replicate 5	Replicate 6	Replicate 7	Replicate 8
Control (WT)	136.27	167.164	166.25	195.38	240.86	140.9	178.78	134.78
Saracatinib (WT)	63.63	89.13	78.84	61.76	37.78	25.77	40.5	36.36
Control (DN)	164.77	229.23	238.667	225.39	128.72	195.833	144.55	205.405
Saracatinib (DN)	98.79	113.92	45.161	91.304	60	22.22	50.925	59.4333

Figure 2F

Percentage of Infected Cells (RPE)	24 hr					
	Replicate 1	Replicate 2	Replicate 3	Replicate 4	Replicate 5	Replicate 6
Control	29.83	31.03	26.31	32.63	23.72	29.94
Saracatinib	9.52	10	7.377	12.6	12.35	12.903

Tachyzoites per 100 Cells (RPE)	24 hr					
	Replicate 1	Replicate 2	Replicate 3	Replicate 4	Replicate 5	Replicate 6
Control	114.737	102.419	109.48	90.78	140.11	156.28
Saracatinib	34.45	27.778	29.1667	25.4	61.04	50

Figure 2G

Percentage of Infected Cells (mHEVc)	24 hr					
	Replicate 1	Replicate 2	Replicate 3	Replicate 4	Replicate 5	Replicate 6
Control (Ctr shRNA)	25.1	29	26.1	27.4	26	28
Saracatinib (Ctr shRNA)	10	9.1	10.5	7	8	10
Control (Src shRNA)	12.1	10	14	9	7	8
Saracatinib (Src shRNA)	13	8.5	11.7	9	8	9

Tachyzoites per 100 Cells (mHEVc)	24 hr					
	Replicate 1	Replicate 2	Replicate 3	Replicate 4	Replicate 5	Replicate 6
Control (Ctr shRNA)	148	171.7	156	164	151	171
Saracatinib (Ctr shRNA)	60	54.1	62.2	42.7	56	52
Control (Src shRNA)	72.6	62	82.5	56	60	51
Saracatinib (Src shRNA)	80	50.2	71.4	55	55	62

Figure 3B

LC3 Accumulation (CHO)	Replicate 1	Replicate 2	Replicate 3	Replicate 4	Replicate 5	Replicate 6
Ctr siRNA	12	3.84	3.703	3.84	12.5	8.33
Src siRNA	53.125	50	46.4285	44	63.63	45.45

LC3 Accumulation (CHO)	Replicate 1	Replicate 2	Replicate 3	Replicate 4	Replicate 5	Replicate 6
Ctr	16	12	3.8	7.4	16	20
Saracatinib	52	44	62.96	68	62	84

Figure 3C

LC3 Accumulation (WT EGFR Brian Endothelial Cells)	Replicate 1	Replicate 2	Replicate 3	Replicate 4	Replicate 5	Replicate 6
Ctr siRNA	3.125	3.33	3.33	4	11.76	10
Src siRNA	44.4444	52	56.25	52	35.7	50

LC3 Accumulation (DN EGFR Brian Endothelial Cells)	Replicate 1	Replicate 2	Replicate 3	Replicate 4	Replicate 5	Replicate 6
Ctr	9.67	7.14	6.66	7.14	3.57	6.89
Saracatinib	51.85	48	68	69.23	45.16	52.94

Figure 3E

LAMP1 Accumulation (CHO)	Replicate 1	Replicate 2	Replicate 3	Replicate 4	Replicate 5	Replicate 6
Ctr siRNA	7.408	3.704	13.889	12	7.14	14.28
Src siRNA	46.85	42.8	42.5	42.105	46.875	38.7096

LAMP1 Accumulation (CHO)	Replicate 1	Replicate 2	Replicate 3	Replicate 4	Replicate 5	Replicate 6
Ctr	4	0	4	4	4	0
Saracatinib	76	80	64	60	50	42.857

Figure 3F

LAMP1 Accumulation (WT EGFR Brian Endothelial Cells)	Replicate 1	Replicate 2	Replicate 3	Replicate 4	Replicate 5	Replicate 6
Ctr siRNA	7.41	4	11.11	12	9.09	7.14
Src siRNA	51.85	57.69	77.78	64	47	50

LAMP1 Accumulation (DN EGFR Brian Endothelial Cells)	Replicate 1	Replicate 2	Replicate 3	Replicate 4	Replicate 5	Replicate 6
Ctr	7.14	6.89	12	8	13.33	6.67
Saracatinib	76	72.413	50	56	41.667	50

Figure 3G

Densitometry Values for Western (CHO)	Src (Compared to Actin)	
	Replicate 1	Replicate 2
Ctr siRNA	1	0.49025
Src siRNA	1	0.584696

Percentage of Infected Cells (CHO)	24 hr					
	Replicate 1	Replicate 2	Replicate 3	Replicate 4	Replicate 5	Replicate 6
Control (Ctr siRNA)	22.7272	26.08	27.27	26.415	29.76	25
Saracatinib (Ctr siRNA)	8.6956	14.28	6.667	7.41	12.79	9.21
Control (ULK1 siRNA)	26.923	30.7692	16.66	12.903	29.82	28.205
Saracatinib (ULK1 siRNA)	29.1666	28.125	13.043	11.764	27.58	25

Tachyzoites per 100 Cells (CHO)	24 hr					
	Replicate 1	Replicate 2	Replicate 3	Replicate 4	Replicate 5	Replicate 6
Control (Ctr siRNA)	145.45	169.56	150	162.26	88.095	95.45
Saracatinib (Ctr siRNA)	60.86	90.476	40	48	33.72	22.36
Control (ULK1 siRNA)	157.69	188.46	102.0833	77.419	89.47	80.76
Saracatinib (ULK1 siRNA)	200	168.75	84.05	67.64	54.022	73.91

Figure 3H

Percentage of Infected Cells (WT EGFR Brain Endothelial Cells)	24 hr					
	Replicate 1	Replicate 2	Replicate 3	Replicate 4	Replicate 5	Replicate 6
Control (Ctr siRNA)	39.0244	33.333	28.662	27.127	20.869	19.04
Saracatinib (Ctr siRNA)	15.05	16.27	13.83	12.34	7.92	7.07
Control (ULK1 siRNA)	37.078	35.3659	27.778	24.4	25.58	29.72
Saracatinib (ULK1 siRNA)	38.3721	34.1176	29.9363	31.3559	28.78	23.43

Tachyzoites per 100 Cells (WT EGFR Brain Endothelial Cells)	24 hr					
	Replicate 1	Replicate 2	Replicate 3	Replicate 4	Replicate 5	Replicate 6
Control (Ctr siRNA)	258.53	242.307	194.267	204.78	105.21	109.52
Saracatinib (Ctr siRNA)	66.666	66.279	91.82	87.037	16.83	24.78
Control (ULK1 siRNA)	242.69	307.31	184.0278	198.1707	102.32	132.43
Saracatinib (ULK1 siRNA)	248.83	235.29	229.9363	216.9492	90	76.56

Figure 3I

Percentage of Infected Cells (DN EGFR Brain Endothelial Cells)	24 hr					
	Replicate 1	Replicate 2	Replicate 3	Replicate 4	Replicate 5	Replicate 6
Control (Ctr siRNA)	36.1538	28.889	34.375	33.33	31.944	26.25
Saracatinib (Ctr siRNA)	13.58	15.29	13.125	5.5	4.58	7.5
Control (ULK1 siRNA)	35.35	34.65	25	30.76923	27.027	32.43
Saracatinib (ULK1 siRNA)	39.21	34.88	30.84112	28.97196	26.37	31.746

Tachyzoites per 100 Cells (DN EGFR Brain Endothelial Cells)	24 hr					
	Replicate 1	Replicate 2	Replicate 3	Replicate 4	Replicate 5	Replicate 6
Control (Ctr siRNA)	220.76	138.88	212.5	190	120.833	112.5
Saracatinib (Ctr siRNA)	57.64	72.83	53.125	39.44	33.58	27.067
Control (ULK1 siRNA)	282.82	264.35	185.4167	162.2378	151.35	181.081
Saracatinib (ULK1 siRNA)	288.235	284.88	236.4486	190.6542	132.96	196.82

Figure 3J

Percentage of Infected Cells (CHO)	24 hr					
	Replicate 1	Replicate 2	Replicate 3	Replicate 4	Replicate 5	Replicate 6
Control (Vehicle)	20.83	21.73	22.71	20.339	18.33	27.38
Saracatinib (Vehicle)	4.65	4.444	9.79	10.94	8.53	7.22
Control (Leupeptin/Pepstatin)	21.0526	20	17.029	16.9742	25.77	26.26
Saracatinib (Leupeptin/Pepstatin)	21.2121	19.35	18.21	20.31	26.667	22.68

Tachyzoites per 100 Cells (CHO)	24 hr					
	Replicate 1	Replicate 2	Replicate 3	Replicate 4	Replicate 5	Replicate 6
Control (Vehicle)	154.1667	152.1739	130.4	118.98	65	66.67
Saracatinib (Vehicle)	32.558	26.667	48.601	59.245	17.07	12.048
Control (Leupeptin/Pepstatin)	163.157	140	129.347	103.69	62.88	62.62
Saracatinib (Leupeptin/Pepstatin)	142.42	135.48	106.785	117.1875	64	59.79

Figure 3K

Percentage of Infected Cells (WT EGFR Brain Endothelial Cells)	24 hr					
	Replicate 1	Replicate 2	Replicate 3	Replicate 4	Replicate 5	Replicate 6
Control (Vehicle)	31.25	32.307	27.94	29.167	35.185	28.48
Saracatinib (Vehicle)	19.23	14.7	14.49	12.65	6.82	11.965
Control (Leupeptin/Pepstatin)	25	30	29.93	28.865	32.394	30.693
Saracatinib (Leupeptin/Pepstatin)	29.54	29.1667	32.25	27.39	33.68	27.35

Tachyzoites per 100 Cells (WT EGFR Brain Endothelial Cells)	24 hr					
	Replicate 1	Replicate 2	Replicate 3	Replicate 4	Replicate 5	Replicate 6
Control (Vehicle)	166.25	195.38	180.147	170.138	172.22	195.15
Saracatinib (Vehicle)	61.764	78.84	62.31	46.83	25.78	46.153
Control (Leupeptin/Pepstatin)	118.18	127.5	142.8571	130.9278	192.95	196.039
Saracatinib (Leupeptin/Pepstatin)	129.54	187.5	204.8387	141.0959	137.89	164.102

Figure 3L

Percentage of Infected Cells (DN EGFR Brain Endothelial Cells)	24 hr					
	Replicate 1	Replicate 2	Replicate 3	Replicate 4	Replicate 5	Replicate 6
Control (Vehicle)	36	38.095	35.93	26.923	25.58	20.98
Saracatinib (Vehicle)	11.29	18.84	12.78	8.88	10.74	9.52
Control (Leupeptin/Pepstatin)	30.5	38.33	31.15	29.92	22.22	22.95
Saracatinib (Leupeptin/Pepstatin)	34	31.034	30	31.182	25.58	25.39

Tachyzoites per 100 Cells (DN EGFR Brain Endothelial Cells)	24 hr					
	Replicate 1	Replicate 2	Replicate 3	Replicate 4	Replicate 5	Replicate 6
Control (Vehicle)	238.666	225.39	203.9063	200	138.37	150.61
Saracatinib (Vehicle)	45.161	91.304	61.65	34.07407	51.23	38.095
Control (Leupeptin/Pepstatin)	181.3559	225	261.59	213	89.81	118.03
Saracatinib (Leupeptin/Pepstatin)	170	131.0344	157.5	190.33	119.76	126.98

Figure 4A

Densitometry Values for Western	p-Akt (Compared to Akt)		
	**Replicate 1**	**Replicate 2**	**Replicate 3**
Uninfected	1	1	1
Infected DMSO Treated	2.83	6.66	1.46
Saracatinib DMSO Treated	1.019	4.022	0.608

Figure 4B

Densitometry Values for Western	p-Akt (Compared to Akt)	
	**Replicate 1**	**Replicate 2**
WT Akt	1	1
CA Akt	126.79	117.37

Percentage of Infected Cells (CHO)	24 hr					
	**Replicate 1**	**Replicate 2**	**Replicate 3**	**Replicate 4**	**Replicate 5**	**Replicate 6**
Control (WT Akt)	28.07	30.158	28.947	27.957	23.42	22.13
Saracatinib (WT Akt)	13.58	9.47	11.864	8.6957	11.702	17.529
Control (CA Akt)	25	26.667	30.159	30.556	27.48	24.749
Saracatinib (CA Akt)	27.58	29.31	28.125	29.06	28.315	30.708

Tachyzoites per 100 Cells (CHO)	24 hr					
	**Replicate 1**	**Replicate 2**	**Replicate 3**	**Replicate 4**	**Replicate 5**	**Replicate 6**
Control (WT Akt)	66.66	52.38	55.263	67.742	86.713	76.67
Saracatinib (WT Akt)	12.6315	27.16	24.576	28.261	40.78	44.62
Control (CA Akt)	53.33	72.05	84.722	65.079	100	99.33
Saracatinib (CA Akt)	75.86	70.689	75.86	84.375	90.68	96.062

Figure 4C

Densitometry Values for Western	p-Akt (Compared to Akt)	
	**Replicate 1**	**Replicate 2**
WT Akt	1	1
CA Akt	7.83	20.53

Percentage of Infected Cells (WT EGFR Brain Endothelial (	24 hr					
	**Replicate 1**	**Replicate 2**	**Replicate 3**	**Replicate 4**	**Replicate 5**	**Replicate 6**
Control (WT Akt)	29.22	28.019	30.71895	33.65385	36.67	34.375
Saracatinib (WT Akt)	10.95	10	6.432749	10.25641	15.55	21.83
Control (CA Akt)	27.108	23.31	31.34328	29.86111	32.55	36.58
Saracatinib (CA Akt)	22.973	24.6667	31.03448	29.11392	38.095	37.755

Tachyzoites per 100 Cells (WT EGFR Brain Endothelial Ce	24 hr					
	**Replicate 1**	**Replicate 2**	**Replicate 3**	**Replicate 4**	**Replicate 5**	**Replicate 6**
Control (WT Akt)	130.519	146.86	169.9346	137.5	374.44	332.81
Saracatinib (WT Akt)	54.79	36	14.03509	33.33333	118.39	102.22
Control (CA Akt)	136.145	128.221	139.5522	104.1667	270.93	362.195
Saracatinib (CA Akt)	106	125.676	108.9655	93.67089	309.52	295.91

Figure 5A

Densitometry Values for Western	p-PI3K (Compared to PI3K)		
	Replicate 1	Replicate 2	Replicate 3
Uninfected	1	1	1
Ctr siRNA Infected	1.598845	2.340148	1.153148
Src siRNA Infected	0.548409	1.180148	0.350479

Densitometry Values for Western	p-PI3K (Compared to PI3K)		
	Replicate 1	Replicate 2	Replicate 3
Uninfected	1	1	1
Infected DMSO Treated	1.607101	1.395682	2.435732
Saracatinib DMSO Treated	0.148201	0.246561	0.491465

Figure 5B

Accumulation p-Akt					
	Replicate 1	Replicate 2	Replicate 3	Replicate 4	Replicate 5
DMSO	55	62.5	31.03448276	40	41.17
Saracatinib	4	3.703703704	0	11.11	7.69

Figure 5C

Densitometry Values for Western	p-PTEN (Compared to PTEN)		
	Replicate 1	Replicate 2	Replicate 3
Uninfected	1	1	1
Ctr siRNA Infected	4.926292	4.057334	6.337946
Src siRNA Infected	1.779295	1.556367	3.501627

Densitometry Values for Western	p-PTEN S380/T382/383(Compared to PTEN)		
	Replicate 1	Replicate 2	Replicate 3
Uninfected	1	1	1
Infected DMSO Treated	4.266036	2.689337	7.728182
Saracatinib DMSO Treated	0.572968	1.242321	2.709143

Densitometry Values for Western	p-PTEN Y240 (Compared to PTEN)	
	Replicate 1	Replicate 2
Uninfected	1	1
Infected DMSO Treated	2.781401	1.426499
Saracatinib DMSO Treated	0.92686	0.275963

Figure 5D

Densitometry Values for Western	PTEN (Compared to Src)	
	Replicate 1	Replicate 2
Uninfected	1	1
Infected DMSO Treated	3.063774	1.568536
Saracatinib DMSO Treated	1.987952	0.997611

Figure 5E

Accumulation PTEN						
	Replicate 1	Replicate 2	Replicate 3	Replicate 4	Replicate 5	Replicate 6
DMSO	10	3	11.36	10.71	13.33	8.69
Saracatinib	64	67.8	48.38	40.9	42.857	43.75

Figure 5F

Densitometry Values for Western (CHO)	PTEN (Compared to Actin)	
	Replicate 1	Replicate 2
Ctr siRNA	1	1
PTEN siRNA	0.18586	0.584156

Percentage of Infected Cells (CHO)	24 hr					
	Replicate 1	Replicate 2	Replicate 3	Replicate 4	Replicate 5	Replicate 6
Control (Ctr siRNA)	26.43	27.53	32.608	32.173	25	21.81
Saracatinib (Ctr siRNA)	11.49	11.23	13.13	16.04	7.69	8.91
Control (PTEN siRNA)	26.92	27.27	30	28.85	31.86	22.97
Saracatinib (PTEN siRNA)	30	26.3889	28.07	30	24	33.33

Tachyzoites per 100 Cells (CHO)	24 hr					
	Replicate 1	Replicate 2	Replicate 3	Replicate 4	Replicate 5	Replicate 6
Control (Ctr siRNA)	93.1	100	185.5	191.3	133.33	134.54
Saracatinib (Ctr siRNA)	24.13	34.83	54.0145	70.37	16.78	20.79
Control (PTEN siRNA)	70.51	78.78	151	111.4	184.61	167.56
Saracatinib (PTEN siRNA)	99	98.611	93.859	126.92	108	102.564

Figure 6A

Histopathology Scores for Eye: 4 weeks Post-Infection	Disruption of Architecture	Perivascular Inflammation	Vitreal Inflammation
Replicate 1	3	2	2
Replicate 2	2	2	1
Replicate 3	1	2	1
Replicate 4	3	3	2
Replicate 5	2	1	1
Replicate 6	2	2	2
Replicate 7	2	2	2
Replicate 8	3	2	2

Figure 6B

Histopathology Scores for Brain: 4 weeks Post-Infection	Diffuse Inflammation	Perivascular Inflammation	Microglial Nodules
Replicate 1	2	3	3
Replicate 2	2	2	1
Replicate 3	2	2	2
Replicate 4	3	2	2
Replicate 5	3	2	3
Replicate 6	2	2	2
Replicate 7	1	1	1
Replicate 8	3	3	2

Figure 6C

Histopathology Scores for Eye: Disruption of Architecture	Control	TMP-SMX	Saracatinib 10 mg/kg	Saracatinib 15 mg/kg
Replicate 1	2	2	0	2
Replicate 2	4	1	1	1
Replicate 3	2	1	0	1
Replicate 4	3	2	1	0
Replicate 5	2	1	1	0
Replicate 6	2		1	1
Replicate 7	3		1	2
Replicate 8	3		2	2
Replicate 9	3		2	
Replicate 10	1		2	
Replicate 11	4		2	
Replicate 12	4		2	
Replicate 13	2		0	
Replicate 14	2		1	
Replicate 15	4		1	
Replicate 16	3			
Replicate 17	3			

Histopathology Scores for Eye: Perivascular Inflammation	Control	TMP-SMX	Saracatinib 10 mg/kg	Saracatinib 15 mg/kg
Replicate 1	2	1	0	1
Replicate 2	2	1	0	0
Replicate 3	2	1	0	0
Replicate 4	2	2	0	0
Replicate 5	2	1	0	0
Replicate 6	1		2	1
Replicate 7	3		1	0
Replicate 8	2		1	0
Replicate 9	3		2	
Replicate 10	0		2	
Replicate 11	2		0	
Replicate 12	3		1	
Replicate 13	1		0	
Replicate 14	2		0	
Replicate 15	3		0	
Replicate 16	1			
Replicate 17	2			

Histopathology Scores for Eye: Vitreal Inflammation	Control	TMP-SMX	Saracatinib 10 mg/kg	Saracatinib 15 mg/kg
Replicate 1	0	1	0	0
Replicate 2	1	0	0	0
Replicate 3	2	0	0	0
Replicate 4	1	1	0	0
Replicate 5	1	0	0	0
Replicate 6	0		0	0
Replicate 7	3		0	0
Replicate 8	2		0	0
Replicate 9	2		0	
Replicate 10	0		0	
Replicate 11	2		0	
Replicate 12	2		0	
Replicate 13	2		0	
Replicate 14	0		0	
Replicate 15	3		0	
Replicate 16	0			
Replicate 17	2			

Figure 6D

Histopathology Scores for Brain: Diffuse Inflammation	Control	TMP-SMX	Saracatinib 10 mg/kg	Saracatinib 15 mg/kg
Replicate 1	2	1	0	0
Replicate 2	4	0	1	0
Replicate 3	4	1	0	0
Replicate 4	2	0	0	0
Replicate 5	1	2	1	0
Replicate 6	3		0	0
Replicate 7	2		0	0
Replicate 8	2		0	0
Replicate 9	3		0	
Replicate 10	3		0	
Replicate 11	3		0	
Replicate 12	2		0	
Replicate 13	3		0	
Replicate 14	3		0	
Replicate 15	2		0	
Replicate 16			1	
Replicate 17			2	

Histopathology Scores for Brain: Perivascular Inflammation	Control	TMP-SMX	Saracatinib 10 mg/kg	Saracatinib 15 mg/kg
Replicate 1	2	1	1	0
Replicate 2	3	1	1	0
Replicate 3	2	1	0	0
Replicate 4	2	0	0	0
Replicate 5	1	1	1	0
Replicate 6	2		0	0
Replicate 7	2		0	0
Replicate 8	3		2	0
Replicate 9	2		0	
Replicate 10	3		0	
Replicate 11	3		1	
Replicate 12	2		0	
Replicate 13	3		0	
Replicate 14	3		0	
Replicate 15	3		0	
Replicate 16			1	
Replicate 17			1	

Histopathology Scores for Brain:Microglial Nodules	Control	TMP-SMX	Saracatinib 10 mg/kg	Saracatinib 15 mg/kg
Replicate 1	2	2	2	0
Replicate 2	4	1	1	1
Replicate 3	4	2	1	0
Replicate 4	2	1	0	1
Replicate 5	2	1	2	1
Replicate 6	3		1	0
Replicate 7	2		1	1
Replicate 8	2		1	0
Replicate 9	2		1	
Replicate 10	3		1	
Replicate 11	3		2	
Replicate 12	2		1	
Replicate 13	2		1	
Replicate 14	2		1	
Replicate 15	3		1	
Replicate 16			2	
Replicate 17			2	

Figure 6E

B1 gene (Eye)	Control	TMP-SMX	Saracatinib 10 mg/kg	Saracatinib 15 mg/kg
Replicate 1	1.45	0.54232914	0.08	0.000013
Replicate 2	1.587	1.20913229	0.407	0.00000376
Replicate 3	1	1.425801687	0.6745	0.00000983
Replicate 4	1.87	0.577518206	0.057	0.0000236
Replicate 5	1		0.27	0.118434849
Replicate 6	0.170855009		0.208	2.46762E-05
Replicate 7	1		0.08707	0.016146394
Replicate 8	0.999998845		1.03362E-05	0.000837552
Replicate 9	0.580101264		5.38468E-07	0.000120029
Replicate 10	0.056158764		2.17431E-05	0.000104706
Replicate 11	0.160565629			

Figure 6F

Tissue Cyst (Brain)	Control	TMP-SMX	Saracatinib 10 mg/kg	Saracatinib 15 mg/kg
Replicate 1	1870	2860	720	220
Replicate 2	2100	2625	270	345
Replicate 3	1320	1470	1260	1470
Replicate 4	1530	2640	880	700
Replicate 5	2250	1800	945	475
Replicate 6	2660		660	460
Replicate 7	2660		708	720
Replicate 8	2240		1760	768
Replicate 9	2160		735	
Replicate 10	2600			
Replicate 11	2070			

Figure 6G

IFNg	Eye		Brain	
	Control	Saracatinib	Control	Saracatinib
Replicate 1	0.049	0.57	2.042710164	0.540064934
Replicate 2	0.21	1.96	1	0.153863879
Replicate 3	2.4149	0.17	0.366934694	0.166804878
Replicate 4	1	0.349	1.144587972	0.224088648
Replicate 5	0.059177217	0.7598	1.424382443	0.649493381
Replicate 6	1.863914207	0.406987126		
Replicate 7		2.167315134		

TNFa	Eye		Brain	
	Control	Saracatinib	Control	Saracatinib
Replicate 1	2.108273744	0.683862457	0.68445868	0.702259154
Replicate 2	0.483391624	1.113831201	1	0.436113928
Replicate 3	3.944215109	1.6559494	0.412862675	0.155641649
Replicate 4	1	1.683833716	0.787074015	0.319467204
Replicate 5	0.211818289	0.783351191	0.861234032	0.236590733
Replicate 6	1.891911531	2.776268448		

IL-12 p40	Eye		Brain	
	Control	Saracatinib	Control	Saracatinib
Replicate 1	1.321728123	0.593000095	0.37295104	0.410069848
Replicate 2	4.762886406	3.308188579	1	0.422781688
Replicate 3	0.431083626	0.638288244	0.314492449	0.050068091
Replicate 4	3.439150756	0.57278789	0.658043643	0.096374946
Replicate 5	1	0.926597878	0.65234787	0.060539324
Replicate 6	0.049231602	0.836718952		
Replicate 7	0.819198536			

NOS2	Eye		Brain	
	Control	Saracatinib	Control	Saracatinib
Replicate 1	1.528112335	0.822670882	1.03668423	0.98192341
Replicate 2	0.376333701	1.001538533	1	0.678976519
Replicate 3	2.010792455	0.204336257	0.399993321	0.171941557
Replicate 4	1	0.654205356	0.709812562	0.817773412
Replicate 5	0.006571466	1.522061161	1.667303108	0.263901293
Replicate 6	0.330540971	0.036079323		
Replicate 7		0.30406191		

Figure 6H

IFNg	Serum	
	Control	Saracatinib
Replicate 1	213.7228888	341.1065438
Replicate 2	207.2118701	384.7182059
Replicate 3	190.1477899	341.1065438
Replicate 4	184.3549812	380.7710635
Replicate 5	185.6268284	248.6223281
Replicate 6	197.4756515	302.4387004
Replicate 7	221.1975819	247.7691295

TNFa	Serum	
	Control	Saracatinib
Replicate 1	28.6474918	61.4474262
Replicate 2	40.0019886	77.3783165
Replicate 3	87.1772565	53.2553192
Replicate 4	179.704513	31.0176733
Replicate 5	146.15046	34.668965
Replicate 6		30.2867333
Replicate 7		36.652762

IL-12 p40	Serum	
	Control	Saracatinib
Replicate 1	9.92	19.465
Replicate 2	15.745	23.15
Replicate 3	30.61	23.42
Replicate 4	16.16	10.895
Replicate 5	18.135	9.05
Replicate 6	17.13	8.555
Replicate 7		7.835

Figure 6I

T. gondii IgG Levels	Serum	
	Control	Saracatinib
Replicate 1	1.622	1.428
Replicate 2	1.3915	1.445
Replicate 3	1.355	1.3375
Replicate 4	1.46	1.4575
Replicate 5	1.5595	1.404

Figure 6J

Accumulation p-Akt	Replicate 1	Replicate 2	Replicate 3	Replicate 4
DMSO	83.33333333	84.375	70	64.197
Saracatinib	15.38461538	11.11111111	32.97	26.35

Figure 6K

Accumulation PTEN	Replicate 1	Replicate 2	Replicate 3	Replicate 4
DMSO	16.66666667	20	20.79	30.76
Saracatinib	65.2173913	56	70.27	74.87

Figure 7A

Histopathology Scores for Eye: Disruption of Architecture	Beclin +/+		Beclin +/-	
	**Control**	**Saracatinib**	**Control**	**Saracatinib**
Replicate 1	2	0	4	4
Replicate 2	1	0	4	3
Replicate 3	1	0	3	3
Replicate 4	2	0		4
Replicate 5	2	1		
Replicate 6		1		

Histopathology Scores for Eye: Perivascular Inflammation	Beclin +/+		Beclin +/-	
	**Control**	**Saracatinib**	**Control**	**Saracatinib**
Replicate 1	2	0	2	2
Replicate 2	3	0	3	2
Replicate 3	3	0	2	2
Replicate 4	2	0		3
Replicate 5	2	0		
Replicate 6		0		

Histopathology Scores for Eye: Vascular Inflammation	Beclin +/+		Beclin +/-	
	**Control**	**Saracatinib**	**Control**	**Saracatinib**
Replicate 1	1	0	2	2
Replicate 2	1	0	1	2
Replicate 3	1	0	2	2
Replicate 4	2	1		2
Replicate 5	1	0		
Replicate 6		1		

Histopathology Scores for Eye: Diffuse Inflammation	Beclin +/+		Beclin +/-	
	**Control**	**Saracatinib**	**Control**	**Saracatinib**
Replicate 1	1	0	2	2
Replicate 2	0	0	2	2
Replicate 3	0	0	2	2
Replicate 4	0	0		2
Replicate 5	1	0		
Replicate 6		0		

Histopathology Scores for Eye: Perivascular Inflammation	Beclin +/+		Beclin +/-	
	**Control**	**Saracatinib**	**Control**	**Saracatinib**
Replicate 1	2	1	2	3
Replicate 2	2	1	3	2
Replicate 3	1	1	3	2
Replicate 4	2	0		3
Replicate 5	2	0		
Replicate 6		1		

Histopathology Scores for Eye: Microglial Nodules	Beclin +/+		Beclin +/-	
	**Control**	**Saracatinib**	**Control**	**Saracatinib**
Replicate 1	2	1	3	2
Replicate 2	2	1	3	3
Replicate 3	2	1	3	2
Replicate 4	2	0		3
Replicate 5	1	0		
Replicate 6		1		

Figure 7B

B1 gene (Eye)	Beclin +/+		Beclin +/-	
	**Control**	**Saracatinib**	**Control**	**Saracatinib**
Replicate 1	1.4	0.2	2	1.55
Replicate 2	1.7	0.4	1.4	1.8
Replicate 3	1	0.2	1.7	1.4
Replicate 4	1.2	0.3		
Replicate 5	0.7	0.2		
Replicate 6		0.4		

B1 gene (Eye)	Beclin +/+		Beclin +/-	
	**Control**	**Saracatinib**	**Control**	**Saracatinib**
Replicate 1	1.4	0.2	2	1.55
Replicate 2	1.7	0.4	1.4	1.8
Replicate 3	1	0.2	1.7	1.4
Replicate 4	1.2	0.3		
Replicate 5	0.7	0.2		
Replicate 6		0.4		

Tissue Cyst (Brain)	Beclin +/+		Beclin +/-	
	**Control**	**Saracatinib**	**Control**	**Saracatinib**
Replicate 1	3296	1232	3864	3570
Replicate 2	2768	1710	4376	3920
Replicate 3	3367	2112	3300	3588
Replicate 4	4256	1740		3680
Replicate 5	2268	1370		
Replicate 6		1608		

Figure S1

MTT Assay (CHO)	Control	1000	100	10	1	0.1	0.01	0.001
Replicate 1	0.2958	0.2397	0.455	0.3457	0.3575	0.3712	0.3813	0.3741
Replicate 2	0.2417	0.1805	0.3466	0.2306	0.2262	0.2917	0.246	0.2413
Replicate 3	0.202	0	0.4	0.2021	0.4459	0.3868	0.2712	0.4271
Replicate 4	0.6199	0	0.0803	0.5477	0.5361	0.4217	0.3443	0.4079
Replicate 5	0.4466	0	0.026	0.271	0.4216	0.2268	0.5397	0.3715
Replicate 6	0.5234		0.051	0.392	0.42333	0.5522	0.4226	0.1202
Replicate 7	0.33			0.2061	0.2327	0.19	0.15	0.107
Replicate 8	0.321			0.108	0.136	0.155	0.097	0.107
Replicate 9	0.226			0.197	0.155	0.082	0.087	

MTT Assay (ARPE)	Control	1000	100	10	1	0.1	0.01	0.001
Replicate 1	0.4906	0.279	0.4355	0.4445	0.5418	0.2869	0.2843	0.2938
Replicate 2	0.409	0.1566	0.2465	0.1694	0.6611	0.3331	0.3133	0.6186
Replicate 3	0.4823	0.2989	0.4325	0.6883	0.3921	0.4564	0.6021	0.5257
Replicate 4	0.2924	0.038	0.19	0.3643	0.3897	0.3281	0.2891	0.45
Replicate 5	0.336	0	0.181	0.6759	0.3852	0.591	0.508	0.436
Replicate 6	0.164	0	0.134	0.477	0.5809	0.416	0.465	0.444
Replicate 7	0.379			0.497	0.363	0.211	0.307	
Replicate 8				0.399	0.335	0.257	0.385	
Replicate 9					0.382			

Figure S2A

Percentage of Intracellular Parasites	**Replicate 1**	**Replicate 2**	**Replicate**	**Replicate 4**	**Replicate 5**	**Replicate 6**	**Replicate 7**
Control	91	90	86	93	89	96	88
Saracatinib	88	84	81	90	89	92	93

Figure S2B

Percentage of Tachyzoites per Vacuole	**Replicate 1**	**Replicate 2**	**Replicate**	**Replicate 4**	**Replicate 5**	**Replicate 6**
Control- 2 Tachyzoites/Vacuole	22.4	21.4814815	25.8621	22.0338983	33.0188679	34.3434343
Control- 4 Tachyzoites/Vacuole	30.1886792	25.2525253	29.3103	37.2881356	28	27.4074074
Control- 8 Tachyzoites/Vacuole	42.4	41.4814815	34.4828	33.8983051	30.1886792	39.3939394
Control- 16 Tachyzoites/Vacuole	6.60377358	1.01010101	10.3448	6.77966102	7.2	9.62962963
Saracatinib- 2 Tachyzoites/Vacuole	25	22.2222222	29.4118	19.2982456	26.119403	14.5833333
Saracatinib- 4 Tachyzoites/Vacuole	23.1343284	30.2083333	22.9167	23.9316239	41.1764706	31.5789474
Saracatinib- 8 Tachyzoites/Vacuole	40.625	47.8632479	27.9412	43.8596491	41.0447761	44.7916667
Saracatinib- 16 Tachyzoites/Vacuole	9.70149254	10.4166667	1.47059	5.26315789	11.4583333	5.98290598

Figure S3A

Percentage of Infected Cells (MEFs)			
		2 hr	
	**Replicate 1**	**Replicate 2**	**Replicate 3**
WT	27.6	24.4	22.1
SYF	31.5	26.2	24.3
SYF + Src	21	23.2	19.9
		24 hr	
	**Replicate 1**	**Replicate 2**	**Replicate 3**
WT	25.7	20.2	19.5
SYF	10.6	8.5	11.9
SYF + Src	24.1	25.3	20.8

Tachyzoites per 100 cells (MEFs)			
		24 hr	
	**Replicate 1**	**Replicate 2**	**Replicate 3**
WT	112.2	107.1	117.6
SYF	42	49	57
SYF + Src	121.2	129	132

Figure S3B

Percentage of Infected Cells (CHO)				
		2 hr		
	**Replicate 1**	**Replicate 2**	**Replicate 3**	**Replicate 4**
Control	23.392	21.739	27.612	29.452
Imatinib 0.3 µM	22.619	23.75	31.214	26.563
Imatinib 1 µM	16.667	25.714	28.767	33.333
	24 hr			
	**Replicate 1**	**Replicate 2**	**Replicate 3**	**Replicate 4**
Control	28.682	28.745	25.94	28.019
Imatinib 0.3 µM	26.531	27.876	24.39	27.619
Imatinib 1 µM	29.958	31.222	30.189	27.072

Tachyzoites per 100 cells (CHO)				
		24 hr		
	**Replicate 1**	**Replicate 2**	**Replicate 3**	**Replicate 4**
Control	144.19	155.06	119.17	158.45
Imatinib 0.3 µM	152.65	160.62	129.76	161.43
Imatinib 1 µM	108.86	132.58	188.68	151.38

Figure S4A

Percentage of Infected Cells (mHEVc)	24 hr			
	**Replicate 1**	**Replicate 2**	**Replicate 3**	**Replicate 4**
Ctr shRNA (Ctr siRNA)	28.947	29.23077	27.52809	27.7027
Src shRNA (Ctr siRNA)	11.33	7.222	8.08	9.87
Ctr shRNA (ULK1 siRNA)	29.93631	29.62963	32.94	24.615
Src shRNA (ULK1 siRNA)	28.48	25.675	27.604	22.44898

Tachyzoites per 100 Cells (mHEVc)	24 hr			
	**Replicate 1**	**Replicate 2**	**Replicate 3**	
Ctr shRNA (Ctr siRNA)	156.31	123.0769	114.6067	122.2973
Src shRNA (Ctr siRNA)	50	32.778	52.52	39.506
Ctr shRNA (ULK1 siRNA)	157.3248	164.8148	142.3529	138.4615
Src shRNA (ULK1 siRNA)	100	109.4595	112.5	120.408

Figure S4B

Percentage of Infected Cells (mHEVc)	24 hr			
	**Replicate 1**	**Replicate 2**	**Replicate 3**	**Replicate 4**
Ctr shRNA (WT Akt)	29.09091	29.19708	25.52	23.71
Src shRNA (WT Akt)	13.125	11.82796	9.21	9.375
Ctr shRNA (CA Akt)	31.69014	29.19255	32.967	29.03226
Src shRNA (CA Akt)	22.41379	26.2069	31.41026	27.442

Tachyzoites per 100 Cells (mHEVc)	24 hr			
	**Replicate 1**	**Replicate 2**	**Replicate 3**	
Ctr shRNA (WT Akt)	129.0909	130.6569	123.4375	164.1026
Src shRNA (WT Akt)	53.125	38.17204	42.76	46.35417
Ctr shRNA (CA Akt)	175.3521	154.6584	180.2198	158.7097
Src shRNA (CA Akt)	68.96552	116.5517	129.4872	143.2558

Figure S5

Densitometry Values for Tissue	Src (Compared to Actin)	
	**Replicate 1**	**Replicate 2**
Brain	1	1.007
Retina	1.406	1.57
Liver	0.473	0.513
Spleen	0.286	0.236
Lung	0.64	0.88

Figure S6

Tissue Cyst (Brain)	4 wks post infection
Replicate 1	1440
Replicate 2	1680
Replicate 3	1937
Replicate 4	1800
Replicate 5	2397
Replicate 6	1494
Replicate 7	2163
Replicate 8	2300
Replicate 9	1730
Replicate 10	1500

Figure S7A

Histopathology Scores for Eye: Disruption of Architecture		
	**PBS**	**Dexamethasone**
Replicate 1	2	4
Replicate 2	2	2
Replicate 3	1	3
Replicate 4	3	3
Replicate 5	1	3
Replicate 6	1	3
Replicate 7	1	4
Replicate 8	2	4

Histopathology Scores for Eye: Perivascular Inflammation		
	**PBS**	**Dexamethasone**
Replicate 1	2	2
Replicate 2	2	2
Replicate 3	1	2
Replicate 4	2	3
Replicate 5	1	2
Replicate 6	1	2
Replicate 7	1	2
Replicate 8	2	3

Histopathology Scores for Eye: Vitreal Inflammation		
	**PBS**	**Dexamethasone**
Replicate 1	2	2
Replicate 2	1	2
Replicate 3	1	1
Replicate 4	2	3
Replicate 5	2	2
Replicate 6	2	2
Replicate 7	1	2
Replicate 8	2	3

Figure S7B

Histopathology Scores for Brain: Diffuse Inflammation		
	**PBS**	**Dexamethasone**
Replicate 1	1	4
Replicate 2	2	3
Replicate 3	2	3
Replicate 4	3	3
Replicate 5	2	3
Replicate 6	2	4
Replicate 7	2	3
Replicate 8	3	3

Histopathology Scores for Brain: Perivascular Inflammation		
	**PBS**	**Dexamethasone**
Replicate 1	3	3
Replicate 2	2	2
Replicate 3	3	3
Replicate 4	2	3
Replicate 5	2	3
Replicate 6	2	3
Replicate 7	2	3
Replicate 8	3	3

Histopathology Scores for Brain:Microglial Nodules		
	**PBS**	**Dexamethasone**
Replicate 1	3	4
Replicate 2	2	3
Replicate 3	2	2
Replicate 4	2	3
Replicate 5	3	4
Replicate 6	2	3
Replicate 7	2	3
Replicate 8	2	3

Figure S7C

Histopathology Scores for Eye: Disruption of Architecture		
**Dexamethasone +**	**DMSO**	**Saracatinib**
Replicate 1	4	1
Replicate 2	3	2
Replicate 3	2	1
Replicate 4	3	1
Replicate 5	3	1
Replicate 6	3	1
Replicate 7	4	2
Replicate 8	3	2
Replicate 9	2	2
Replicate 10	3	
Replicate 11	3	
Replicate 12	4	
Replicate 13	3	

Histopathology Scores for Eye: Perivascular Inflammation		
**Dexamethasone +**	**DMSO**	**Saracatinib**
Replicate 1	2	1
Replicate 2	2	2
Replicate 3	2	1
Replicate 4	3	0
Replicate 5	2	1
Replicate 6	2	1
Replicate 7	2	0
Replicate 8	3	2
Replicate 9	2	0
Replicate 10	1	
Replicate 11	3	
Replicate 12	2	
Replicate 13	3	

Histopathology Scores for Eye: Vitreal Inflammation		
**Dexamethasone +**	**DMSO**	**Saracatinib**
Replicate 1	2	0
Replicate 2	2	2
Replicate 3	1	0
Replicate 4	3	0
Replicate 5	2	1
Replicate 6	2	1
Replicate 7	2	0
Replicate 8	3	1
Replicate 9	2	1
Replicate 10	2	
Replicate 11	3	
Replicate 12	2	
Replicate 13	3	

Figure S7D

Histopathology Scores for Brain: Diffuse Inflammation		
**Dexamethasone +**	**DMSO**	**Saracatinib**
Replicate 1	3	1
Replicate 2	4	1
Replicate 3	3	2
Replicate 4	3	1
Replicate 5	2	0
Replicate 6	3	2
Replicate 7	3	1
Replicate 8		0
Replicate 9		1
Replicate 10		1

Histopathology Scores for Brain: Perivascular Inflammation		
**Dexamethasone +**	**DMSO**	**Saracatinib**
Replicate 1	3	1
Replicate 2	3	1
Replicate 3	3	1
Replicate 4	3	1
Replicate 5	2	0
Replicate 6	3	1
Replicate 7	3	0
Replicate 8	2	0
Replicate 9		1
Replicate 10		0

Histopathology Scores for Brain:Microglial Nodules		
**Dexamethasone +**	**DMSO**	**Saracatinib**
Replicate 1	4	2
Replicate 2	3	2
Replicate 3	3	1
Replicate 4	3	1
Replicate 5	3	1
Replicate 6	3	2
Replicate 7	3	1
Replicate 8	2	1
Replicate 9		2
Replicate 10		2

Figure S7E

B1 gene (Eye)			
	**PBS**	**Dexamethasone**	**Dexamethasone + Saracatinib**
Replicate 1	1.792768141	5.146332051	0.36665281
Replicate 2	2.386592927	1.680694157	0.000802098
Replicate 3	2.692075677	1.979606929	0.017301159
Replicate 4	1	1.206151339	0.144539019
Replicate 5	2.225917059	1.667956904	0.049097625
Replicate 6		1.896608127	0.005013194
Replicate 7		1.502776719	

Figure S6F

Tissue Cyst (Brain)			
	**PBS**	**Dexamethasone**	**Dexamethasone + Saracatinib**
Replicate 1	1222	2100	320
Replicate 2	1995	2800	1210
Replicate 3	1680	1995	840
Replicate 4	2160	3675	1110
Replicate 5		1980	800
Replicate 6		2400	630
Replicate 7		2300	1080
Replicate 8		5400	512.5
Replicate 9		3080	1200
Replicate 10		1100	1100
Replicate 11		3220	
Replicate 12		1125	
Replicate 13		1430	
